# Identification of new risk factors for rolandic epilepsy: CNV at Xp22.31 and alterations at cholinergic synapses

**DOI:** 10.1136/jmedgenet-2018-105319

**Published:** 2018-05-22

**Authors:** Laura Addis, William Sproviero, Sanjeev V Thomas, Roberto H Caraballo, Stephen J Newhouse, Kumudini Gomez, Elaine Hughes, Maria Kinali, David McCormick, Siobhan Hannan, Silvia Cossu, Jacqueline Taylor, Cigdem I Akman, Steven M Wolf, David E Mandelbaum, Rajesh Gupta, Rick A van der Spek, Dario Pruna, Deb K Pal

**Affiliations:** 1 Department of Basic and Clinical Neuroscience, Institute of Psychiatry, Psychology and Neuroscience, Maurice Wohl Clinical Neuroscience Institute, King’s College London, London, UK; 2 Neuroscience Discovery Research, Eli Lilly and Company, Surrey, UK; 3 Department of Neurology, Sree Chitra Tirunal Institute for Medical Sciences and Technology, Trivandrum, Kerala, India; 4 Department of Neurology, Hospital de Pediatría Prof. Dr. J.P. Garrahan, Combate de los Pozos 1881, Buenos Aires, Argentina; 5 Department of Biostatistics and Health Informatics, Institute of Psychiatry, Psychology & Neuroscience, King’s College London, London, UK; 6 NIHR Biomedical Research Centre at South London and Maudsley NHS Foundation Trust, Bethlem Royal Hospital, Beckenham, UK; 7 Farr Institute of Health Informatics Research, UCL Institute of Health Informatics, University College London, London, UK; 8 Department of Paediatrics, University Hospital Lewisham, Lewisham and Greenwich NHS Trust, London, UK; 9 Department of Paediatric Neurosciences, Evelina London Children’s Hospital, St Thomas’ Hospital, London, UK; 10 Department of Paediatric Neurology, Chelsea and Westminster Hospital, London, UK; 11 Neurosurgery Unit, Neuroscience and Neurorehabilitation Department, Bambino Gesù Children Hospital, Rome, Italy; 12 Neurology Unit, Pediatric Hospital A. Cao, Brotzu Hospital Trust, Cagliari, Italy; 13 Barnet and Chase Farm Hospitals, London, UK; 14 Division of Pediatric Neurology, College of Physicians and Surgeons of Columbia University, New York City, New York, USA; 15 Department of Neurology, Mount Sinai Health System, New York City, New York, USA; 16 Departments of Pediatrics, Alpert Medical School of Brown University, Providence, Rhode Island, USA; 17 Department of Paediatrics, Tunbridge Wells Hospital, Pembury, UK; 18 Department of Neurology, Brain Center Rudolf Magnus, University Medical Center Utrecht, Utrecht, The Netherlands

**Keywords:** copy-number, developmental, genome-wide, epilepsy and seizures

## Abstract

**Background:**

Rolandic epilepsy (RE) is the most common genetic childhood epilepsy, consisting of focal, nocturnal seizures and frequent neurodevelopmental impairments in speech, language, literacy and attention. A complex genetic aetiology is presumed in most, with monogenic mutations in *GRIN2A* accounting for >5% of cases.

**Objective:**

To identify rare, causal CNV in patients with RE.

**Methods:**

We used high-density SNP arrays to analyse the presence of rare CNVs in 186 patients with RE from the UK, the USA, Sardinia, Argentina and Kerala, India.

**Results:**

We identified 84 patients with one or more rare CNVs, and, within this group, 14 (7.5%) with recurrent risk factor CNVs and 15 (8.0%) with likely pathogenic CNVs. Nine patients carried recurrent hotspot CNVs including at 16p13.11 and 1p36, with the most striking finding that four individuals (three from Sardinia) carried a duplication, and one a deletion, at Xp22.31. Five patients with RE carried a rare CNV that disrupted genes associated with other epilepsies (*KCTD7*, *ARHGEF15*, *CACNA2D1, GRIN2A* and *ARHGEF4*), and 17 cases carried CNVs that disrupted genes associated with other neurological conditions or that are involved in neuronal signalling/development. Network analysis of disrupted genes with high brain expression identified significant enrichment in pathways of the cholinergic synapse, guanine-exchange factor activation and the mammalian target of rapamycin.

**Conclusion:**

Our results provide a CNV profile of an ethnically diverse cohort of patients with RE, uncovering new areas of research focus, and emphasise the importance of studying non-western European populations in oligogenic disorders to uncover a full picture of risk variation.

## Introduction

With an incidence of 1/2500, rolandic epilepsy (RE) or benign epilepsy with centrotemporal spikes (MIM:117100) is the most common genetic childhood epilepsy.^1 2^ Children experience focal seizures usually in sleep that affect the vocal tract, with sensorimotor symptoms that progress to the tongue, mouth and face, resulting in hypersalivation and speech arrest. The characteristic EEG abnormality is blunt centrotemporal spikes (CTS) typically with frontal positivity and sleep activation. RE starts at a mean of 7 years, and there is a spontaneous remission of seizures during adolescence.^3 4^ Neurodevelopmental comorbidities such as speech sound disorder, language impairment, reading disability, migraine and attention impairment are common both in children with RE and their families^5–7^; however, the prognosis for these conditions is less clear.

The underlying genetic basis of RE is still largely unknown. Both an autosomal-dominant model of inheritance of the EEG trait CTS^8^ and a complex mode of inheritance for seizure risk^9^ have been suggested. Loci for CTS have been identified at *ELP4-PAX6* on 11p13^10 11^ and at 15q13 (*CHRNA7)*.^12^ Independent replication of *ELP4* SNP association with CTS was not achieved.^13^ However, it has subsequently been shown that it is variation at a micro-RNA seed region within the 3′ untranslated region of *PAX6* which increases the risk of CTS.^14^ Rare mutations in RE cases, with and without neonatal convulsions, were first found in potassium channels *KCNQ2* and *KCNQ3.* 15 With the advent of large-scale exome sequencing, mutations in five further genes are now shown to be rare risk factors; the NMDA receptor *GRIN2A*; neuronal splicing regulators *RBFOX1* and *RBFOX3; GABRG2—*a gamma-aminobutyric acid (GABA) type A receptor; and a repressor of the mammalian target of rapamycin (mTOR) complex 1 signalling pathway, *DEPDC5*.^16–21^

CNV is a well-known risk factor in both generalised and focal epilepsies, and is also an important cause of epileptic encephalopathies (EE).^22–24^ Both recurrent ‘hotspot’ and rare inherited and *de novo* CNVs are beginning to explain the diverse phenotypes in individual families and across disorders, suggesting a shared molecular basis for several phenotypes. Children with EEs that form part of the epilepsy-aphasia spectrum (of which RE is at the mild end), namely Landau-Kleffner syndrome (LKS) and continuous spike and waves during slow wave sleep (CSWS), carry a heterogeneous mixture of rare CNVs, with enrichment for cell adhesion genes associated with autism and language impairment.^25^ In a small study of 47 patients with either typical or EEG-atypical RE, half carried rare microdeletions and microduplications, some disrupting known causal genes for epilepsy. Two patients in this study with atypical RE also carried the 16p11.2 recurrent duplication.^26^ Motivated by this finding, Reinthaler *et al* undertook a screening of six recurrent CNVs in patients with typical RE and LKS/CSWS/atypical benign partial epilepsy ABPE.^27^ 1.2% of those with RE, and one with LKS, carried the 600 kb 16p11.2 duplication, and one a 110 kb duplication, which was in significant excess compared with controls.

These studies prompted us to carry out a genome-wide investigation of CNV in a large cohort of well-phenotyped, typically presenting patients with RE. We report the presence of both a large number of causal and potentially causal heterogeneous, rare CNVs, as well as recurrent hotspot CNVs in these patients. The most prominent findings are of recurrent duplications and one deletion at Xp22.31, and the network clustering of genes involved in cholinergic synapses and guanine-exchange factor activation.

## Methods

### Study participants

Five groups of patients with typical RE at presentation from different genetic backgrounds (total n=195) were included in this study from the UK (n=41), the USA (n=52), Sardinia (n=62), Kerala, India (n=34), and Argentina (n=6). We also analysed 70 ethnically matched control subjects from Kerala. Written informed consent was obtained from all participating families, both parents and, where appropriate, children/adolescents. All respective local institutional review boards approved the study. In total, 800 control individuals for the network analysis, called NL4, were matched for gender and ethnicity (see below) to the European cases from a population-based study in the Netherlands. Ascertainment and ethical considerations are found in refs.^28 29^

### Case ascertainment and definition

RE probands and their families were prospectively recruited for genetic studies from the US paediatric neurology centres in New York, New Jersey, Pennsylvania, Connecticut, Rhode Island and Massachusetts; from southeastern UK paediatric centres; from the Epilepsy Unit at the Pediatric Hospital, Cagliari Sardinia; from Sree Chitra Institute for Medical Science and Technology, Kerala; and from the Department of Neurology, Hospital de Pediatría Prof. Dr. J.P. Garrahan, Buenos Aires, Argentina. Ascertainment was through the proband. RE cases were enrolled if they met stringent eligibility criteria of at least one witnessed seizure with typical features: nocturnal, simple partial seizures affecting one side of the body, or on alternate sides; oro-facial-pharyngeal sensorimotor symptoms, with speech arrest and hypersalivation; age of onset between 3 and 12 years; no previous epilepsy type; normal global developmental milestones; normal neurological examination; at least one interictal EEG with centrotemporal sharp waves and normal background; and neuroimaging (if performed) that excluded an alternative structural, inflammatory or metabolic cause for the seizures. All participants were evaluated by a physician for associated clinical features. We categorised seizure number into low (<10 lifetime seizures) and high (≥10 lifetime seizures); and antiepileptic drugs (AEDs) used as zero or monotherapy versus two or more.

### Genotyping and CNV detection

We used high-density SNP genotyping arrays to detect the presence of CNVs from genomic DNA: HumanOmniExpress-12 for all the RE cases and Indian controls, and HumanCoreExome-12 V.1-0 for the UK family members as well as the UK cases, which were therefore typed on both arrays (Illumina, USA). NL4 controls were also genotyped on the HumanOmniExpress-12 as detailed in ref.^30^ Arrays were processed according to the manufacturer’s instructions. To minimise false positives, CNVs were called using both the PennCNV software^31^ and the Nexus Copy Number package (BioDiscovery, USA) from signal intensity data after preprocessing in Illumina GenomeStudio Software. For PennCNV, a GC model file was incorporated into the analysis to correct for GC-rich regions that cause genomic waves in signal intensities. The clean_cnv.pl script (https://github.com/WGLab/PennCNV/blob/master/clean_cnv.pl) was also run to merge adjacent CNVs with a gap <20% of the total merged length. The sample-level quality control (QC) criteria used for PennCNV were SD for autosomal log R ratio >0.26, B allele frequency drift of >0.003, waviness factor −0.05 to 0.05, and samples with more than45 CNVs were removed. Cut-offs for CNV calls in PennCNV were for variants that contained >15 consecutive altered SNP probes and were >20 kb in size. In the Nexus software, systematic array correction files were used for the two different arrays to correct for GC bias, and a significance threshold of 1×10^–7^ was applied. The SNP-FAST2 Segmentation algorithm was used for analysis, with homozygous frequency threshold at 0.95, hemizygous loss threshold at −0.23 and single copy gain at 0.13 for the log R ratio. Nine samples were removed from the project because they had a <95% call rate, a probe to probe variability (quality score) of >0.2 or a gender mismatch, leaving n=186. Cut-offs for CNV calls were the same as for PennCNV. CNVs were only taken forward to the next stage of analysis if they were called from both software approaches and, for UK samples, if they were called from both array types. CNVs showing >90% overlap with variants of a frequency of ≥0.1% of the same type, reported in the Database of Genomic Variants (http://projects.tcag.ca/variation/), were considered copy number polymorphisms and were excluded from further analysis, therefore CNVs analysed here are designated as ‘rare’.

### Validation of CNVs

We validated CNVs for the UK cases using the Illumina HumanCoreExome-12 chip. Validation for other cases/family members was performed with real-time quantitative PCR (qPCR) using the Qiagen (USA) Type-it CNV Syber Green Kit according to the manufacturer’s instructions and as in ref.^32^ The ΔΔC_T_ method of relative quantification was used, and the ratio (R) of the copy number change of the gene of interest in the case sample compared with the control sample calculated using R=2^-ΔΔC^T.

### QC for ethnicity matching

A QC was performed separately on both case/family and NL4 control datasets, according to methods previously published.^30^ Both datasets were tested for the presence of related and duplicated samples (pi-hat >0.1). After the QC, the case and control datasets were merged with the HapMap3 dataset and the first four principal components (PCs) were calculated using the EIGENSTRAT method. Individuals of non-European ancestry that scattered >10 SD from the HapMap CEU mean, considering the first four PCs, were removed. After the removal of population outliers, PCs were calculated on an LD-pruned set of SNPs, considering only genotype data from the case and NL4 datasets (MAF cut-off >0.011, based on ref.^33^). Outliers were removed considering a deviation from the stratum mean of the first four PCs of >5 SD (online supplementary tables S1 and S2, and supplementary figure S1).

10.1136/jmedgenet-2018-105319.supp1 Supplementary data

### Network analysis

Network analysis was used to identify if the genes disrupted by the RE CNVs are enriched for certain types of biological function and to identify other genes and pathways that may interact with the disrupted RE genes, giving ideas about potential new avenues of understanding for the disorder. To identify interaction partners, separate gene networks were created in Ingenuity Pathway Analysis (IPA, Qiagen Bioinformatics, Denmark) of CNV disrupted genes with high brain expression from the European ancestry cases or controls. IPA constraints were to include direct and indirect relationships, 70 genes within the network, only experimentally observed data and only endogenous mammalian node types. To identify CNV disrupted genes with high brain expression, we used the BrainSpan database^34^ available from http://www.brainspan.org/static/download.html and genes with average log2 RPKM >4.5 (the top 18%). The genes formed within the IPA network were then used as an input for the enrichment and functional annotation tool Enrichr^35^ (http://amp.pharm.mssm.edu/Enrichr/). Enrichr was used to identify KEGG pathways and GO terms that had significant enrichment (p<0.05, adjusted for the number of tests for the category type). Enrichr carries out computational analysis of the input sets by comparing them to these annotated gene sets representing prior biological knowledge.

In order to identify potential physical connectivity between proteins, a list of all of the genes from the rare CNVs identified in RE patients was uploaded to the Disease Association Protein-Protein Link Evaluator (DAPPLE) V.0.17 within GenePatten (https://genepattern.broadinstitute.org/gp).^36^ DAPPLE extracts seed genes from the uploaded gene list and converts them into proteins which are found in the InWeb database of high-confidence protein–protein interactions. Also, 1000 random networks were generated by permutation to assess if the connectivity of each seed protein with the reference protein–protein interaction network was greater than expected by chance. The most highly enriched genes (corrected p<0.05) were uploaded to IPA for network analysis using the settings as above.

## Results

We studied genome-wide CNV content in a cohort of 186 unrelated patients with RE using high-density SNP genotyping arrays. The patients were from diverse genetic backgrounds; from the UK (n=41), the USA (n=49), Sardinia (n=58), Kerala, India (n=32), and Argentina (n=6), which allowed us to identify additional variants that may differ from those common in mainland European populations. We identified 84 patients (45%) with one or more rare CNVs (frequency <0.1% in control populations from the Database of Genomic Variants (http://projects.tcag.ca/variation/), as well as unreported in 70 Kerala controls): 14 carried CNVs that are recurrent risk factors (7.5%), table 1, and 15 carried CNVs that were likely pathogenic (8.0%), a total n=28, because two patients carried both a pathogenic and a likely pathogenic CNV (table 2). These CNVs were confirmed by qPCR or analysis of ExomeChip data. The manual assignation of potential pathogenicity was based on gene content (exons were always disrupted), CNV size, segregation with disease or *de novo* occurrence, and previous literature on epilepsy and related neurological disorders. The remaining 56/84 participants carried rare CNVs that were of uncertain clinical significance. The mean rare CNV length was 305 kb and median length 155 kb. Also, 10 out of the 14 carriers of risk factor CNVs were female, compared with 35% of the overall cohort. Inheritance could be assessed in most patients from the UK and US cohorts due to the availability of parental DNA (online supplementary figure S2).

**Table 1:** Individuals with rolandic epilepsy (RE) and recurrent risk factor CNVs

Case ID (gender)	Cytoband	CNV coordinates (hg19/B37)	Size (kb)	CNV type (inheritance)	UCSC gene content	Case phenotype	Family phenotype	Hotspot/disease association
S218 (F)	1p36.33	chr1:0–1065691	1065	Dup	*OR4F5, OR4F29 SAMD11, NOC2L, KLHL17, PLEKHN1 (+6 more)*	RE onset 9 years	Cousin FS; mother; migraine	Hotspot/*KLHL17* candidate gene. Seen in AE. Del causes infantile spasms.
SFR (M)	1q21.1	chr1:14595197–145926106	530	Dup	*HFE2, TXNIP, POLR3GL, (+18 more)*	RE onset 9 years	None	Hotspot. ID, DD, epilepsy, dysmorphic features.
JRS (M)	1q21.1q21.2	chr1:145926106–147826789	1900	Dup	*LOC100288142, NBPF10, PDZK1P1, NBPF11, (+17 more)*	RE onset 5 years, freq seizures, ESES, multiple Rx	Mother; migraine	As above.
1050-301 (F)	2q21.1	chr2:131598135–131772974	174	Del (Pat)	*ARHGEF4*	RE onset 9 years, frequent seizures	Maternal grandmother and uncle; MIG, paternal uncle; RD and stutter, aunt; migraine	2q21.1 locus, ID, epilepsy, LI and ADHD.
RK044 (M)	7q11.21	chr7:66048230–66130669	154	Del	*KCTD7*	RE onset 8y	Unknown	Progressive myoclonic epilepsy.
SMJ (F)	7q21.11	chr7:82045382–82148862	103	Dup	*CACNA2D1*	RE onset 10 years, headache	Cousin; grand-mal seizures	West syndrome, epilepsy and ID.
RK011 (F)	16p13.2	chr16:9964443–10080978	116	Del	*GRIN2A*	RE onset 3 years, freq seizures, multiple Rx, RD	Unknown	Genetic focal epilepsies with rolandic spikes.
1012-301 (F)	16p13.11	chr16:14952508–16333313	1380	Del (*de novo*)	*NOMO1, NPIP, NTAN1, PDXDC1, RRN3*, *(+11 more)*	RE onset 3 years	Sister; RD	16p13.11 hotspot. genetic generalised epilepsies, diverse epilepsies.
7083-301 (F)	17p13.1	chr17:8219833–8243565	23	Dup (*de novo*)	*ARHGEF15, ODF4*	RE onset 8 years, RD	Sister; abnormal EEG, RD, SPC; mother; partial epilepsy, migraine.	*ARHGEF15*: epileptic encephalopathy, ID, SD and LI.
S201 (F)	Xp22.31	chrX:6439256–8138035	1698	Dup	*VCX3A, HDHD1, STS, VCX, PNPLA4, VCX2*	RE, onset 9 years	None	Hotspot ID, epilepsy inc. RE, AE, ASD.
S241 (F)	Xp22.31	chrX:6439256–8138035	1698	Dup	*VCX3A, HDHD1, STS, VCX, PNPLA4, VCX2*	RE, onset 7 years	Cousin; probable IFE	As above.
1052-301 (F)	Xp22.31	chrX:6449682–8138035	1688	Del (*de novo*)	*VCX3A, HDHD1, STS, VCX, PNPLA4, VCX2*	RE onset 6 years, SD	Father; depression	As above.
S149 (F)	Xp22.31	chrX:6449682–8138035	1688	Dup	*VCX3A, HDHD1, STS, VCX, PNPLA4, VCX2*	RE onset 5 years	FH of FS	As above.
1039-301 (M)	Xp22.31	chrX:7497771–8138035	640	Dup (*Mat*)	*VCX, PNPLA4, VCX2*	RE onset 7 years, SD, migraine	Father; RD	As above.

Genetic and clinical characteristics of 14 patients with RE and their families who carried a CNV classed as pathogenic.

References can be found in online supplementary table S3.

AE, absence epilepsy; ASD, autism spectrum disorder, DD, developmental delay; ESES, electrical status epilepticus in sleep; F, female; FH, family history; FS, febrile seizures; ID, intellectual disability; IFE, idiopathic focal epilepsy; LI, language impairment; M, male; RD, reading difficulty or dyslexia; Rx, pharmacological treatments; SD, speech disorder; UCSC, University of California, Santa Cruz.

**Table 2:** Individuals with rolandic epilepsy (RE) and potentially pathogenic CNVs

Case ID (gender)	Cytoband	CNV coordinates (hg19/B37)	Size (kb)	CNV type (inheritance)	UCSC gene content	Case phenotype	Family phenotype	Disease association/function
7007-301 (F)	1p31.1	chr1:71414151–71469541	55	Del (Mat)	*PTGER3*	RE onset 10 years	Paternal half-sister; absences, ADHD, SD, RDG	FS. Inflammation pathway gene.
RK029 (M)	1q22	chr1:155492432–155644686	152	Dup	*ASH1L, ASH1L-AS1, MSTO2P, MSTO1, YY1AP1*	RE onset 5 years, RD, ADHD	Unknown	*ASH1L* disruptive variants cause ASD, ID, seizures.
1053-301 (M)	2p12	chr2:79342216–79461716	119	Dup (*de novo*)	*REG1A, REG1P, REG3A, CTNNA2*	RE onset 6 years, frequent seizures	Father; RD, depression, sister; RD, migraine	CTNNA2; cell–cell adhesion, axon guidance, dendrite aborisation. ADHD, SCZ.
S218 (F)	2q34	chr2:212410753–212698130	287	Dup	*ERBB4*	RE onset 9 years	Cousin; FS, mother; migraine	Early myoclonic encephalopathy. Regulates neuronal excitability and plasticity.
7037-301 (F)	4p15.2	chr4:21542615–21566331	23	Del (*de novo*)	*KCNIP4*	RE onset 4 years, RD, ADHD	Mother; RD, migraine	*KCNIP4*; regulates neuronal excitability. ADHD candidate.
S52 (F)	4q22.1–22.2	chr4:93556411–94663992	1107	Dup	*GRID2*	RE onset 10 years, RD, dyscalculia	Cousin; RE	Glutamate receptor delta2. Causes cerebellar ataxia, DD, SD.
1029-301 (F)	5q11.2	chr5:56740642–58265912	1525	Dup (Mat)	*ACTBL2, PLK2, GAPT, RAB3C, PDE4D*	RE onset 6 years	None	*PDE4D* functions in memory. *PLK2* regulates dendritic spine morphology, increases post seizure.
7007-301 (F)	8q13.2	chr8:67998878–68249106	250	Dup (Mat)	*CSPP1, ARFGEF1*	RE onset 10 years	Paternal half-sister; absences, ADHD, SD, RDG	*ARFGEF1* regulates neurite outgrowth and polarity. EE candidate.
SSM (M)	10q21.3	chr10:67609352–67731403	122	Del	*CTNNA3*	RE onset 8 years, freq seizures, multiple Rx, ADHD, LD.	None	Del in EE and ASD. Cell adhesion molecule, stab. dendritic spines.
S38 (M)	10q23.1	chr10:83538813–83667589	128	Dup	*NRG3*	RE onset 7 years	None	SCZ, bipolar disorder, DD, ASD. Pleiotropic neurodevelopment roles.
1041-301 (M)	11q14.1	chr11:84078744–84347934	269	Dup (Mat)	*DLG2*	RE onset 9 years	Pat grandmother; migraine, mat grandmother; depression	Assoc. ASD, DD, bipolar disorder. Encodes PSD-93—binds and controls glutamate receptors.
7021-302 (F)	12q14.3	chr12:67060969–67382547	321	Del (Mat)	*GRIP1*	RE onset 6 years, RD, SD, motor dyspraxia	Mother; ADHD, depression, MIG, sister and brother; MIG, brother RE	Synaptic scaffold protein stabilises glutamate receptors. Increased in epileptic mice. ASD.
1027-301 (M)	15q21.3	chr15:54857821–54924743	66	Del (Mat)	*UNC13C*	RE onset 6 years, RD, SD, ADHD, motor dyspraxia	Maternal aunt and grandfather; LD	Presynaptic protein mediates synaptic vesicle priming and plasticity.
SMJ (F)	15q22.33	chr15:67426523–67454644	28	Del	*SMAD3*	RE onset 10 years, headache	Cousin; grand mal seizures	Pathway regulates synaptogenesis and contributes to seizures in TLE rats.
7034-301 (M)	17q12	chr17:31958395–32931677	973	Dup (Mat)	*ASIC2, CCL2, CCL7, CCL11, CCL8, CCL13, CCL1, C17orf102, TMEM132E*	RE onset 5 years, frequent seizures, multiple Rx, SD, RD.	Mother; FS, migraine; mat uncle and aunt; RD, migraine, FS, mat cousin; RD	*ASIC2*; ion channel, activity terminates seizures.
NVH (M)	20q12	chr20:41049613–41275309	225	Del	*PTPRT*	RE onset 4 years, freq seizures, ESES, multiple Rx	Father; FS, mother; ADHD	Regulates synaptic function and neuronal development.
1027-301 (M)	Xq27.3	chrX:144853754–145033725	179	Del (Mat)	*SLITRK2, TMEM257*	RE onset 6 years, RD, SD, ADHD, motor dyspraxia	Maternal aunt and grandfather; LD	*SLITRK2:* assoc. SCZ, ASD, RD. Controls excitatory synapse formation.

Genetic and clinical characteristics of 15 patients with RE and their families who carried CNVs classed as potentially pathogenic. Two patients, 7007-301 and 1027-301, carried two potentially pathogenic CNVs. S218 and SMJ also carry a pathogenic CNV, table 1.

References can be found in online supplementary table S3.

ASD, autism spectrum disorder; DD, developmental delay; F, female; FH, family history; FS, febrile seizures; ID, intellectual disability; LD, learning difficulties; LI, language impairment; M, male; Mat, maternal; MIG, migraine; Pat, paternal; RD; reading difficulty or dyslexia; SCZ, schizophrenia; SD, speech disorder; UCSC, University of California, Santa Cruz.

### Hotspot CNVs

**Figure 1:**
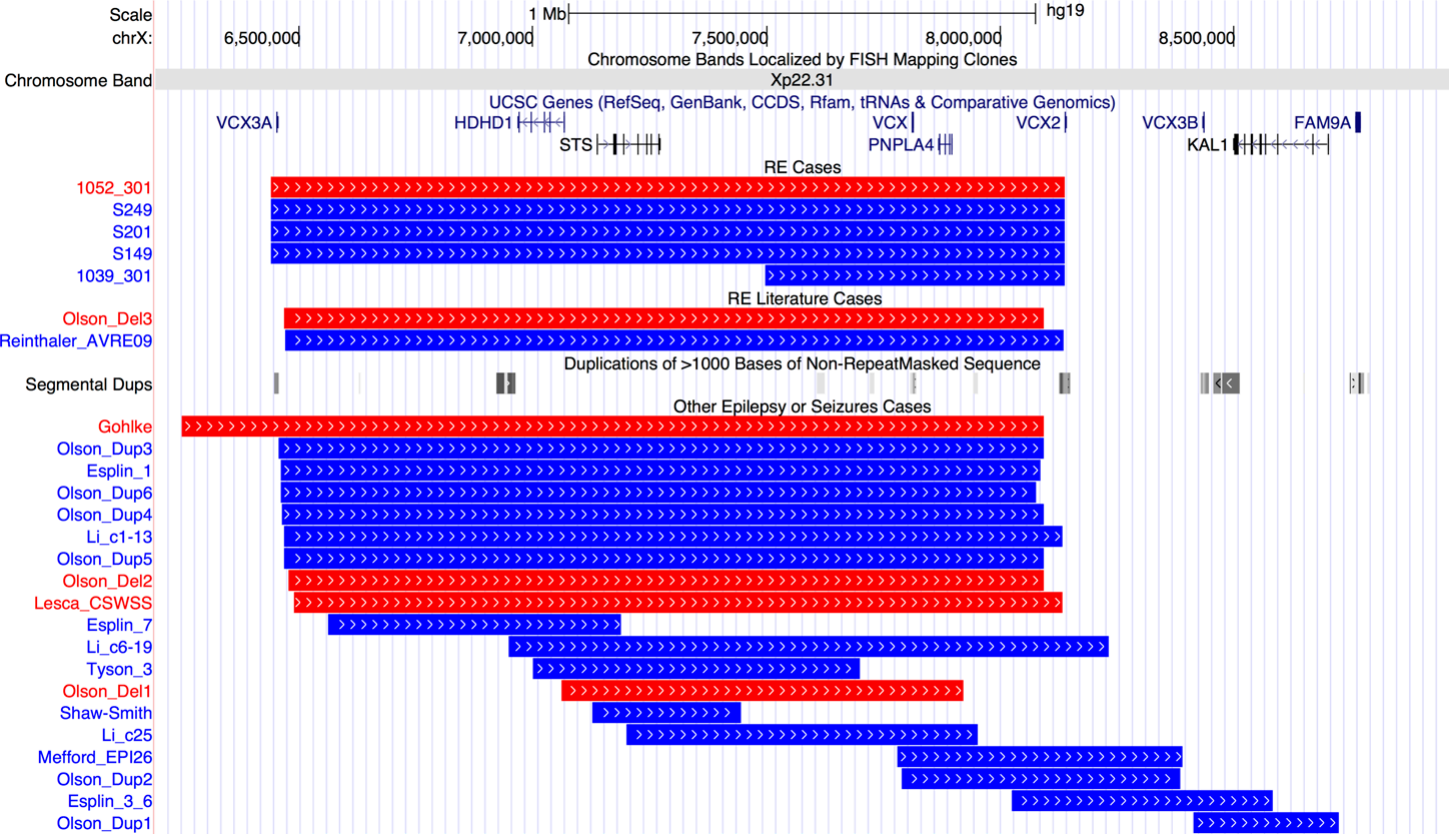
Breakpoints of 5 cases with Xp22.31 hotspot CNVs in our rolandic epilepsy (RE) case series, 2 cases with RE and Xp22.31 CNVs from the literature and 19 further cases with epilepsy or seizures form the literature. Individual IDs or publication references are shown to the left and references are in online supplementary table 3. Blue lines indicate duplications and red lines deletions. Gene positions are shown above the CNVs. Positions of segmental duplication sequence (locus control regions) are shown in the middle of the figure with grey bars. From http://genome.ucsc.edu/, hg19 assembly. UCSC, University of California, Santa Cruz.

Pathogenic CNVs classified as a risk factor for RE include those that are ‘hotspots’—regions of the genome that contain large segmental duplications, allowing non-allelic homologous recombination during cell division. Nine patients carried hotspot CNVs, with the most striking finding that four individuals carried a duplication and one a deletion at Xp22.31 (figure 1). Three duplications and one deletion were at the most frequent breakpoints (6.44–8.14 Mb) and one duplication had a more distal start (7.49–8.14 Mb).

Other hotspot CNVs included a deletion at 16p13.11 and a deletion of the distal 1p36 region, containing the candidate gene *KLHL17.* 37 Two further individuals carried duplications of the 1q21 hotspot. We could assess inheritance for three patients carrying hotspot CNVs (online supplementary figure S2); 16p13.11 deletion occurred *de novo* in patient 1012-301, as did the Xp22.31 deletion in 1052-301. The Xp22.31 duplication in 1039-301 was inherited from his unaffected mother.

### Rare CNVs

Nineteen patients with RE (ie, those from tables 1 and 2 that do not carry hotspot CNVs described earlier, apart from one patient that had both a hotspot and rare CNV) were found to either carry a rare CNV that disrupted genes that are recurrent risk factors for epilepsy (n=5, disrupting *GRIN2A*, *KCTD7*, *ARHGEF15*, *CACNA2D1* and *ARHGEF4*) (table 1), genes that are associated with other neurological conditions or that are involved in neuronal signalling and/or development (table 2). We could assess inheritance and family segregation of these CNVs in 12 of the 19 patients (tables 1 and 2, online supplementary figure S2). Two of the CNVs occurred *de novo*, and 10 were inherited from a parent (two in one patient from the mother), sometimes with a family history of epilepsy or neurodevelopmental disorders.

### Network analysis

To identify interaction partners and potential new pathways disrupted in RE, we created gene networks with IPA using as input the CNV-disrupted genes with high brain expression from the European ancestry cases versus European controls. The top network generated for each dataset (now including genes not within the CNVs) was input into the Enrichr enrichment annotation tool to identify biological meaning, including enrichment for functional terms.^35^ Enrichr uses a modified Fisher’s exact test to calculate if the genes from the network are found more frequently within a gene set library (Gene Ontology) or pathway (KEGG) than the random chance across the genome for that category. It uses the Benjamini-Hochberg false discovery rate to correct for multiple hypotheses. For the European cases, the KEGG pathway of cholinergic synapse was significantly enriched P_Adj_=9.6×10^−7^, and the GABAergic synapse, P_Adj_=6.2×10^− 4^ (online supplementary figures S3 and S4, and table S4). GO molecular function terms were enriched for GTPase and rho guanyl-exchange factor activation, and biological process terms for the positive regulation of apoptotic processes and protein phosphorylation cascades (online supplementary tables S5 and S6). For the controls, no KEGG pathway was enriched, and the only enriched GO terms were molecular function terms that are involved in mRNA and small RNA binding (online supplementary table S7).

**Figure 2:**
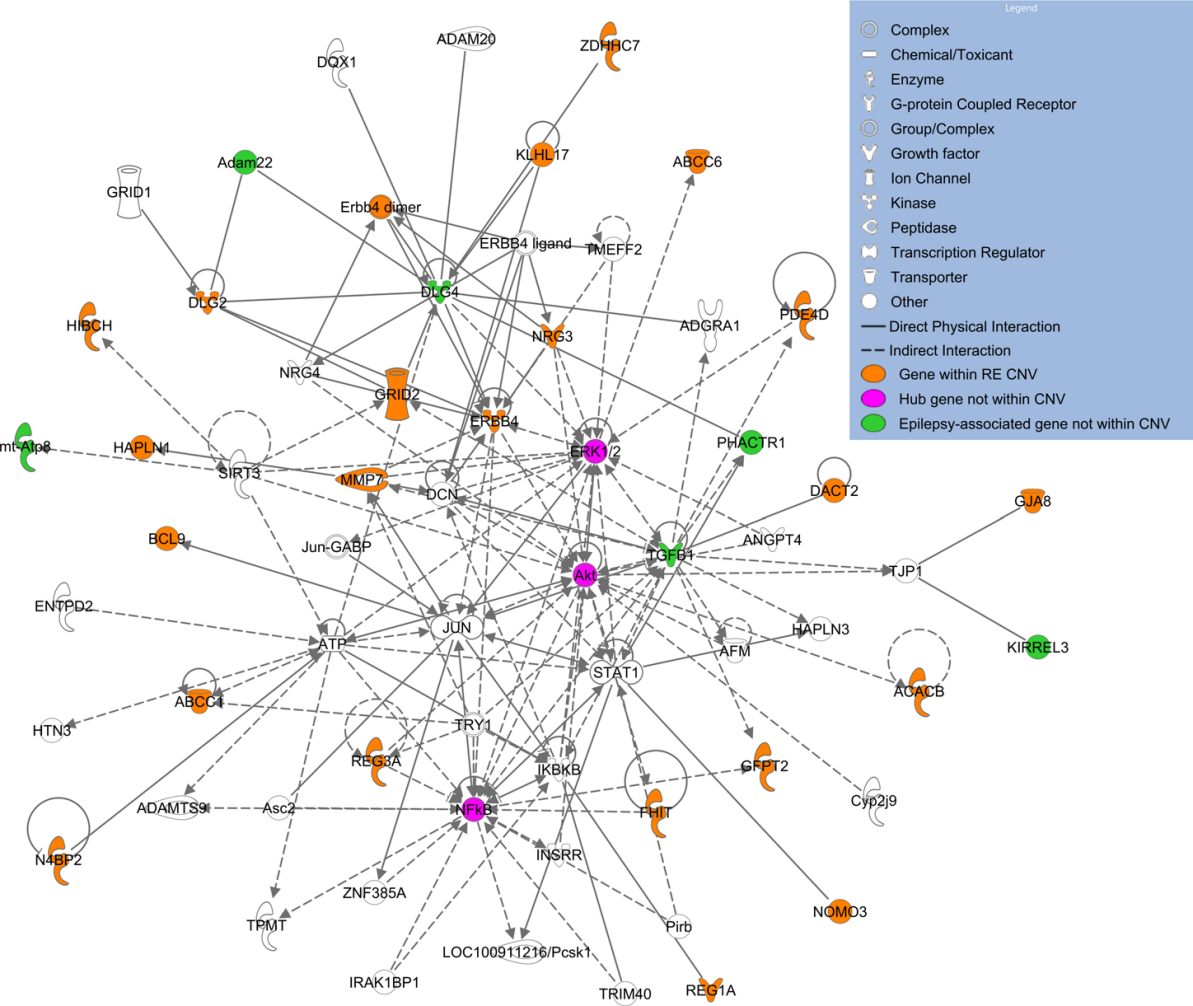
Network created by Ingenuity Pathway Analysis using the top 36 most highly connected genes disrupted by rolandic epilepsy (RE) CNVs as assessed by the Disease Association Protein-Protein Link Evaluator.^36^ Orange indicates a gene within a CNV, pink a hub gene, green an epilepsy-associated gene not found within a CNV and white are genes added by Ingenuity Pathway Analysis during network generation due to direct physical or indirect (eg, via activation) interactions with the input list.

In order to identify significant protein interaction networks that modulate disease risk encoded by disrupted genes identified from all of the patients with RE, we applied DAPPLE.^36^ This tool extracted ‘seed genes’ from the 279 genes in the CNVs using the InWeb database of high-confidence protein–protein interactions. Ten had highly enriched connectivity (p<0.01 corrected for multiple testing over random network generation): *REG1A, CTNNA3*, DLG2*, *HIBCH*, *ABCC6*, CTNNA2*, KLHL17, RPL9*, NEIL2* and NOC2L*. Five of these genes also show high expression in the brain, indicated with an asterisk. A further 26 genes (12 with high brain expression) had corrected p values<0.05. An IPA network formed using these 36 genes as input, but allowed to expand to 70 genes on network formation to identify interaction partners (figure 2) contained three hubs not found within the CNVs themselves. Two hubs, AKT1/2/3 part of the mTOR pathway (mutated in focal cortical dysplasia^38^) and ERK1/2 (also known as MAP kinases 1 and 2), are important regulators of synaptic excitability involved in epilepsy in animal models and human disease.^39^ Six other genes within the network, not found in these RE CNVs, are also epilepsy candidates, indicating the strength of this approach for finding disrupted gene pathways (figure 2).

### Phenotypic correlates

We used two-tailed Fisher’s exact tests for comparison of proportions to analyse the association between the presence of a risk factor or potential risk factor CNV, and either seizure frequency or need for multiple AEDs (table 3). Our results show that the presence of a risk factor or potential risk factor CNV is not associated with high seizure number (>10 lifetime seizures), p=0.3, nor the need for multiple AEDs, p=0.07.

**Table 3:** Numbers of patients with rolandic epilepsy within different seizure and antiepileptic drug (AED) categories

	Patients with no risk/potential risk factor CNVs (n)	Patients with risk or potential risk factor CNVs (n)	Total	P values
<10 lifetime seizures	97	14	111	0.3
>10 lifetime seizures	52	12	64	
0–1 AED	108	14	122	0.07
≥2 AEDs	43	13	56	

(Eleven patients are missing seizure frequency and eight missing AED data).

## Discussion

From this large investigation of genome-wide CNV in children with RE, we identified both recurrent and rare heterogeneous CNVs that contain genes involved in synapse formation, neuronal excitability and synaptic plasticity, axon guidance and neuronal development. Four patients with RE carried CNVs that disrupted genes known to cause other epilepsies; *KCTD7*, *ARHGEF4*, *ARHGEF15* and *CACNA2D1*, expanding the phenotypes associated with these genes. One individual was also found with a breakpoint within the known risk factor gene *GRIN2A*.^16–18^ These, and other pathogenic CNVs, were not associated with seizure number or AED use variables, suggesting that they do not generally result in more severe phenotypes.

The genetic model of RE remains complex: including our data, both exome sequencing and CNV analysis, has identified only a small amount of the overall genetic risk. The heterogeneous nature of RE is underpinned by our study, where only variation at Xp22.31 is recurrent. Out of the 30 rare CNVs identified in patients with RE by Dimassi, only two overlap with our larger cohort; we both identified one patient with a maternally inherited deletion of part of *UNC13C*, and we identified a *de novo* deletion of the 16p13.11 hotspot, whereas Dimassi identified a maternally inherited duplication of the same region.^26^ A heterogeneous mixture of CNVs has also been identified in a cohort of patients with LKS and CSWS^17^, which form the severe end of the epilepsy-aphasia spectrum, with RE at the mild end. These CNVs, as here in RE, often contain genes associated with other neurodevelopmental disorders such as ASD and LI, especially cell adhesion proteins, strengthening the aetiological overlaps between these disorders.

We have identified both *de novo* and inherited CNVs, with several unaffected carrier parents, reflecting the incomplete penetrance that is common in the genetic epilepsies.^23^ Indeed, for other genetic variants associated with RE such as mutations in *GRIN2A*, *GABRG2* and *DEPDC5*, as well as for 16p11.2 duplications, incomplete penetrance is commonly noted.^16–20 27^ This might partly be because mild phenotypes presenting in childhood in earlier generations, such as a single seizure or early difficulties with speech and language may have been forgotten and not reported. In some of these families it is likely that additional, and as yet largely unknown, genetic factors contribute to the risk of developing RE. Indeed, two genetic ‘hits’ are not an uncommon observation in RE. In our study, we identified four patients carrying two rare risk factor CNVs, Dimassi *et al* described 10/47 patients with two rare CNVs^26^ and Reinthaler *et al* 27 identified 1 patient with RE carrying two hotspot CNVs and 1 family with a *de novo* 16p11.2 duplication and an inherited *DEPDC5* mutation. In a further study, one patient with RE was found to carry a *de novo GABRG2* mutation as well as an inherited *GRIN2A* mutation, and a second a paternally inherited 15q11.2 duplication and a maternally inherited *GABRG2* mutation. Candidate gene or exome sequencing of the cohort described in this paper may therefore allow identification of further second hits.

One of the most striking findings of the study is an enrichment of CNV at the Xp22.31 locus in five patients with RE compared with previous reports.^24 27^ Contiguous gene syndromes have long been associated with microdeletions at Xp22.31, the phenotypic nature of which depends on the genes encompassed.^40^ Common phenotypes in those with CNV at Xp22.31 are mental retardation, developmental delay and ichthyosis, with seizures and epilepsy less frequently reported. However, a recent paper has observed epilepsy in 24% of paediatric cases.^24^ Locus control regions (long-range cis-regulatory elements) especially at the distal ends of *VCX3A* and *VCX2* predispose this region to non-alleleic homologous recombination (figure 1).^41^ Genes in the most commonly disrupted region of Xp22.31 (figure 1) are *VCX3A*, which may contribute to mental retardation^42^; *HDHD1*, a phosphatase involved in dephosphorylation of a modified RNA nucleotide^43^; *STS*, encoding a steroid sulfatase which hydrolyses neurosteroids that affect membrane potential and current conductance of the neuron, controlling network excitability and seizure susceptibility; *VCX*, which regulates mRNA translation and neurite outgrowth^44^; *PNPLA4*, which plays a key role in triglyceride hydrolysis and energy metabolism; and *VCX2*, which is not yet well characterised. Thus, there are several genes in the region which could potentially contribute individually, or in an interacting model, to the seizure and neurodevelopmental profile of RE. The differing size and location of smaller CNVs identified in other publications that disrupt only one or two of the genes at Xp22.31 indicates a minimum common region cannot be identified to account for the seizure phenotype, and other factors may also be required. Therefore, while a definitive molecular aetiology cannot be provided at this stage, the addition of five cases with RE expands on the seizure phenotypes associated with the Xp22.31 region, especially in Sardinian patients.

It is of note that we did not observe any duplications at the 16p11.2 locus previously reported in around 1% of patients with RE^26 27^ (although the two patients reported in ref.^26^ had atypical RE). However, only one Xp22.31 duplication was reported in these two previous cohorts. This could indicate that the differing genetic backgrounds of the participants may play a role in the contrasting enrichment patterns. The three ‘typical breakpoint’ deletion patients at Xp22.31 described here are unrelated Sardinians. Sardinia is more isolated genetically than other parts of the Mediterranean, and may constitute a pre-Indo-European population. However, there is also substantial heterogeneity within Sardinia itself due to its internal geography. The population of southern Sardinia appears to cluster somewhat with European populations with regard to allele distributions, whereas that of northern Sardinia is highly differentiated and determined by contributions of several ethnic groups, potentially including northern African and Middle Eastern origins.^45^ Thus our work highlights the importance of studying populations with different genetic backgrounds to fully identify the risk factors in oligogenic disorders such as RE. We also did not find deletions at 15q13.3 or 15q11.2 and only one deletion at 16p13.11. These hotspot rearrangements have been associated with genetic generalised epilepsies (GGEs), but not with genetic focal epilepsies, a distinction that is further strengthened here.^22 46 47^

Network analysis of the RE CNV genes identified several interesting pathways that may indicate new risk factors (cholinergic synapse, guanine exchange factors) and give further strength to those already implicated (mTOR pathway, MAP kinases). Acetylcholine (ACh) acts as a neuromodulator within the brain, causing changes in neuronal excitability and synaptic plasticity, altering release of neurotransmitters and coordinating the firing of groups of neurons.^48^ The enrichment for brain expressed genes within cholinergic synapses introduces the regulation of ACh signalling as a potential new pathogenic pathway. Indeed, fluctuating levels of ACh during different sleep states correlate with periods of increased seizure susceptibility.^49^ Alterations to both presynaptic and postsynaptic responses to ACh, as seen here, may influence network excitability at these critical time points during sleep in patients with RE. This theory will now require further work in both cellular and animal models, and correlations with patient sleep EEG for confirmation.

Genes with high brain expression and those that stimulate the exchange of GDP for GTP, guanine nucleotide exchange factors, (GEFs), to activate GTPases, were also significantly enriched in European cases. GEFs regulate many aspects of cytoskeletal organisation, such as the morphogenesis and plasticity of dendritic spines and the induction of long-term potentiation, as well as vesicle transport and the regulation of excitatory synapse development. Indeed, GEFs have already been implicated in learning disability with epilepsy^50^ and shown to have function-altering mutations in epileptic encephalopathy,^51 52^ indicating their potential importance as an avenue to explore in RE causality.

We were limited in our study by the low number of matched controls from the Kerala region, which meant we were not able to carry out a case–control analysis for this cohort. However, we have reported the CNVs descriptively if they were not present in the matched controls or other databases. Another limitation is that we did not have parental DNA for many of the RE cases to assess CNV inheritance. This would have aided in the putative assignment of pathogenicity, as those that arise *de novo*, or segregate with affectedness, would more likely predispose to the epilepsy.

In summary, we show that rare CNVs may play a pathogenic role in a significant proportion of children with RE, although the model of genetic risk still requires elucidation. Network analysis of genes with high brain expression from this ethnically diverse cohort suggests the involvement of new molecular pathways in rolandic epilepsy. The prevalence and nature of recurrent CNVs in RE can differ by population, but also clearly differ from those involved in GGEs. Aside from a few rare monogenic cases, it is likely that the majority of RE is explained by interactions between sequence and CNV, and this hypothesis could be addressed in future large-scale studies.
