# Supplementary material for: Identification of new risk factors for rolandic epilepsy: CNV at Xp22.31 and alterations at cholinergic synapses
Source: J Med Genet. 2018 May 22;55(9):607–16. doi: 10.1136/jmedgenet-2018-105319 (PMC6119347; doi:10.1136/jmedgenet-2018-105319)
Supplement: Supplementary data [file jmedgenet-2018-105319supp001.pdf]

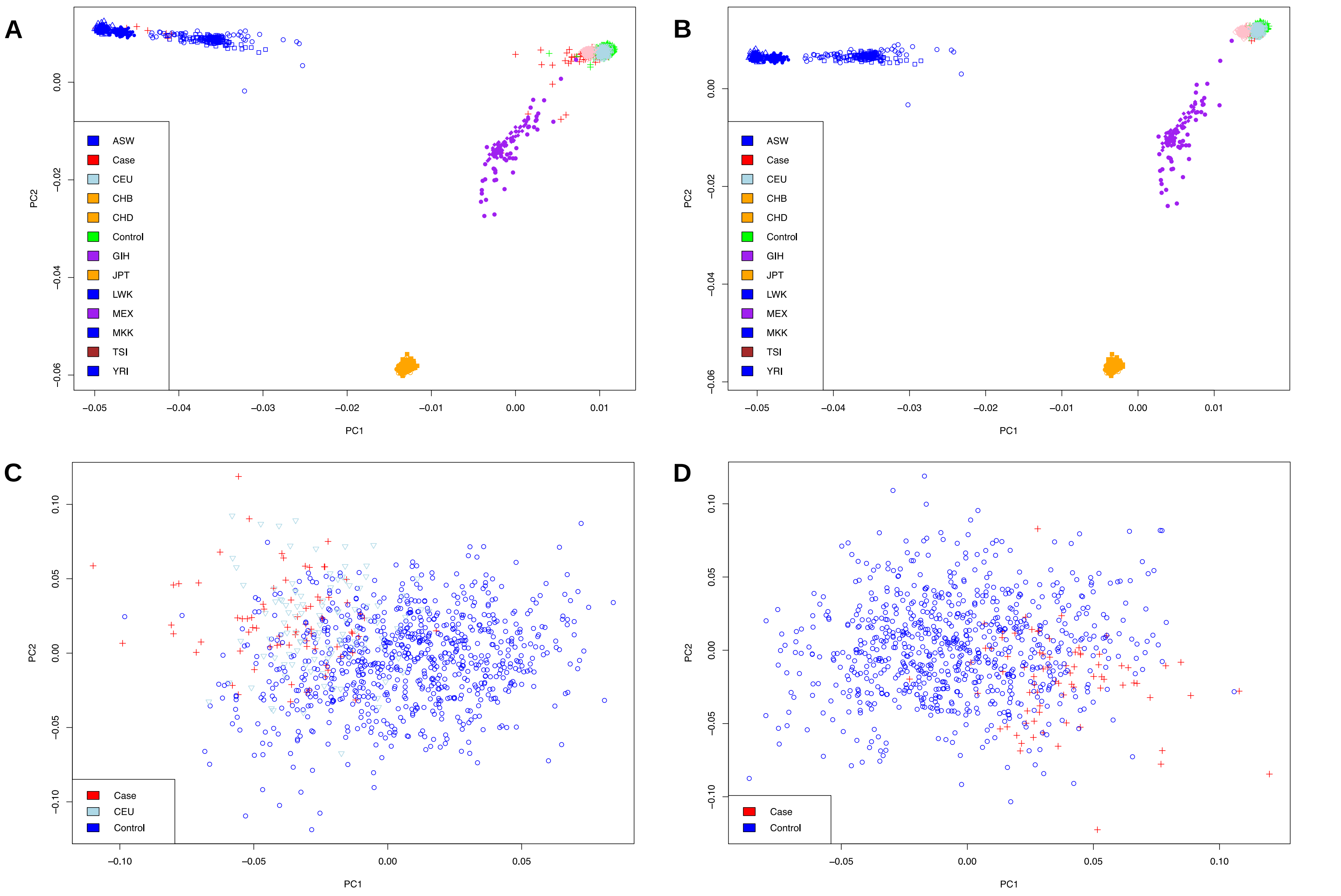

Supplementary Figure S1(**A**) The first two principal components were calculated combining HapMap 3 individuals with the RE (Case) and NL4 (Control) datasets. (**B**) Individuals of non-European ancestry were removed if they were ±10 s.d. from the HapMap CEU mean considering

the first four Principal Components (PCs). A subsequent removal of samples deviating by more than 5 s.d. from the stratum mean of the first two PCs was also performed (Calculated on Genotyped data only). (**C**) Individuals remaining after removing population outliers resulted in a homogenous European sample. (**D**) Individuals remaining after removing population outliers resulting in a homogenous group between the RE cases and NL4 datasets.

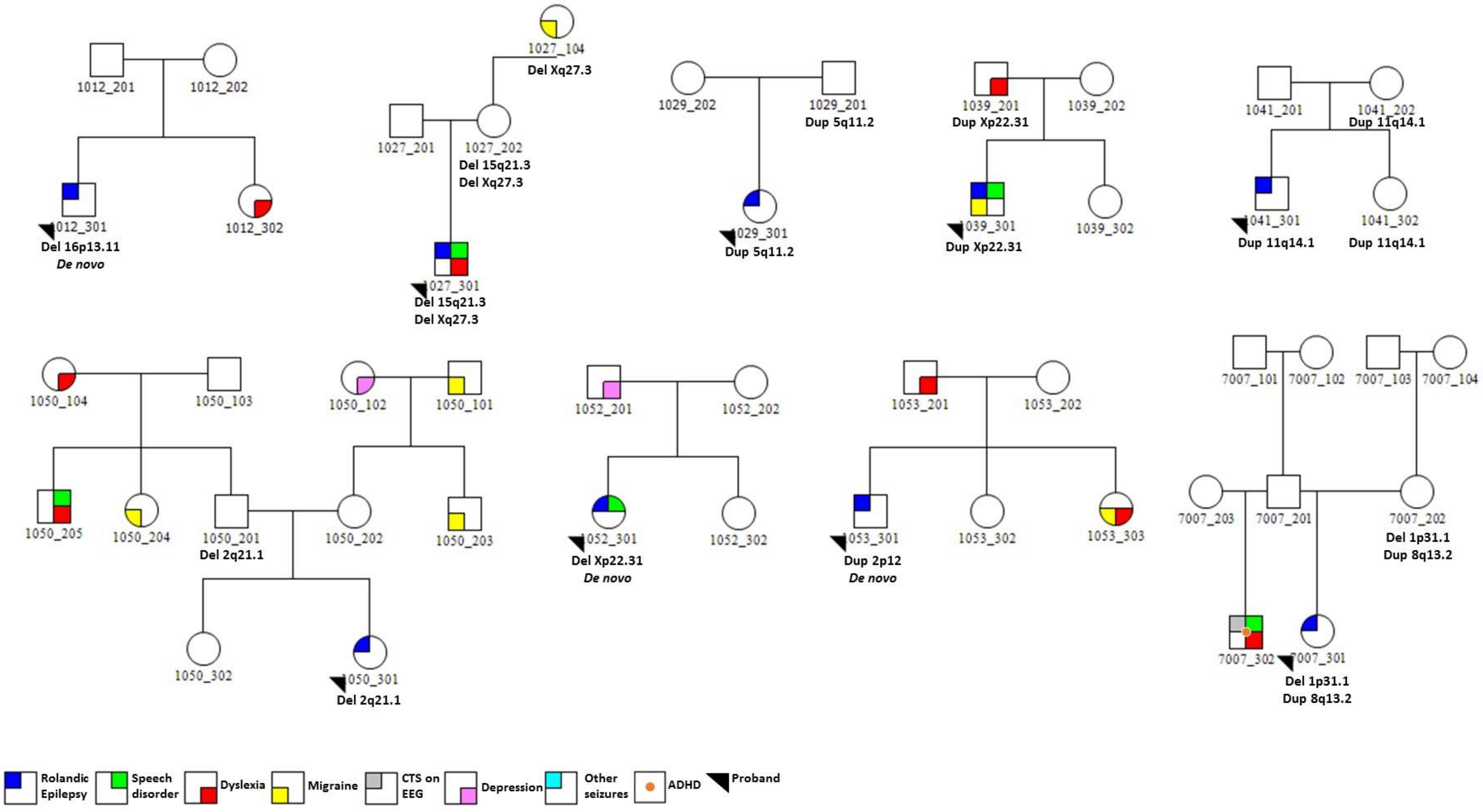

Supplementary Figure S2 (cont. on next page). Pedigree diagrams of families with RE and hotpost or rare CNVs where inheritance information was available. Circles indicate females, and squares males. Details of CNVs are found below individuals that carry that CNV.

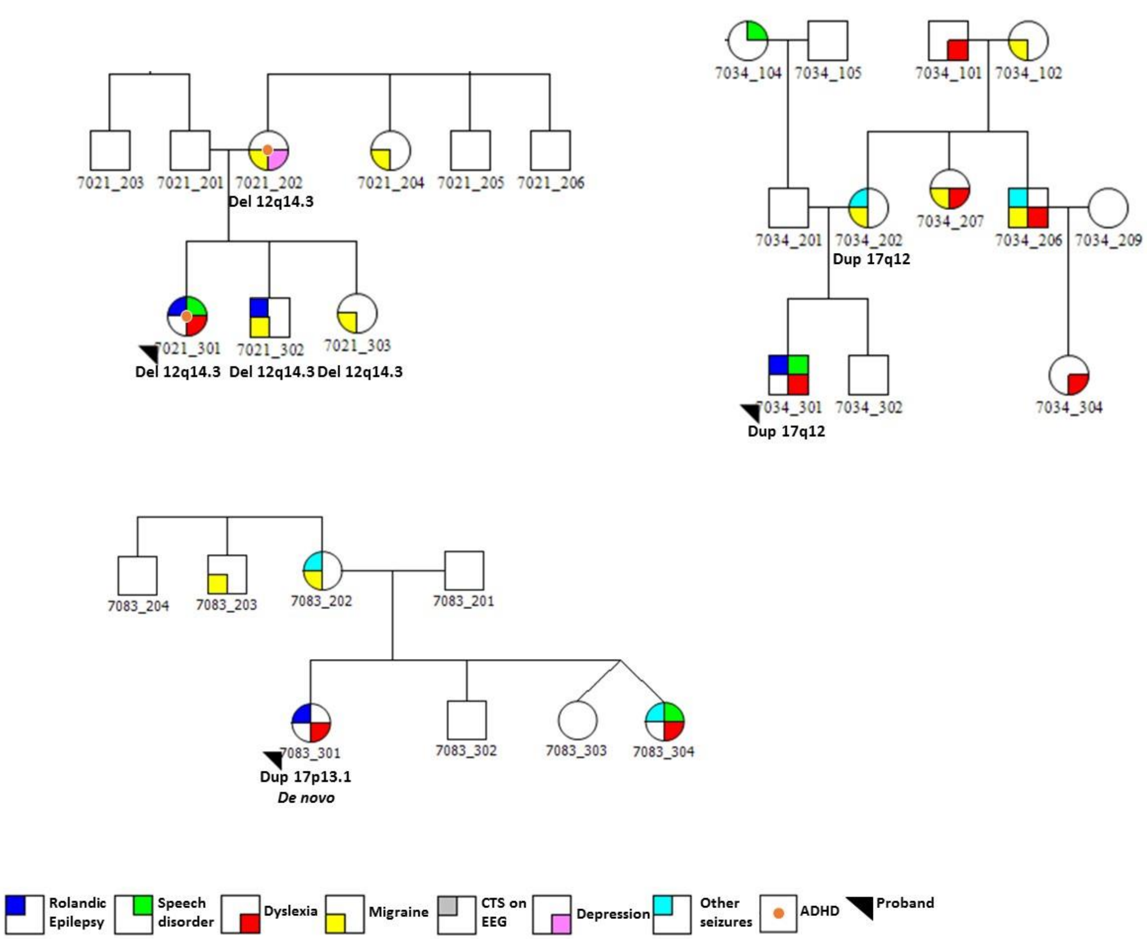
7021_203
7021_201
7021_202
Del 12q14.3
7021_204
7021_205
7021_206
7021_301
7021_302
7021_303
Del 12q14.3
Del 12q14.3
Del 12q14.3
7034_104
7034_105
7034_101
7034_102
7034_201
7034_202
Dup 17q12
7034_207
7034_206
7034_209
7034_301
7034_302
7034_304
Dup 17q12
7037_104
7037_101
7037_102
7037_103
7037_201
7037_202
7037_205
7037_203
7037_301
7037_302
Del 4p15.2
De novo
7083_204
7083_203
7083_202
7083_201
7083_301
7083_302
7083_303
7083_304
Dup 17p13.1
De novo
Rolandic Epilepsy
Speech disorder
Dyslexia
Migraine
CTS on EEG
Depression
Other seizures
ADHD
Proband

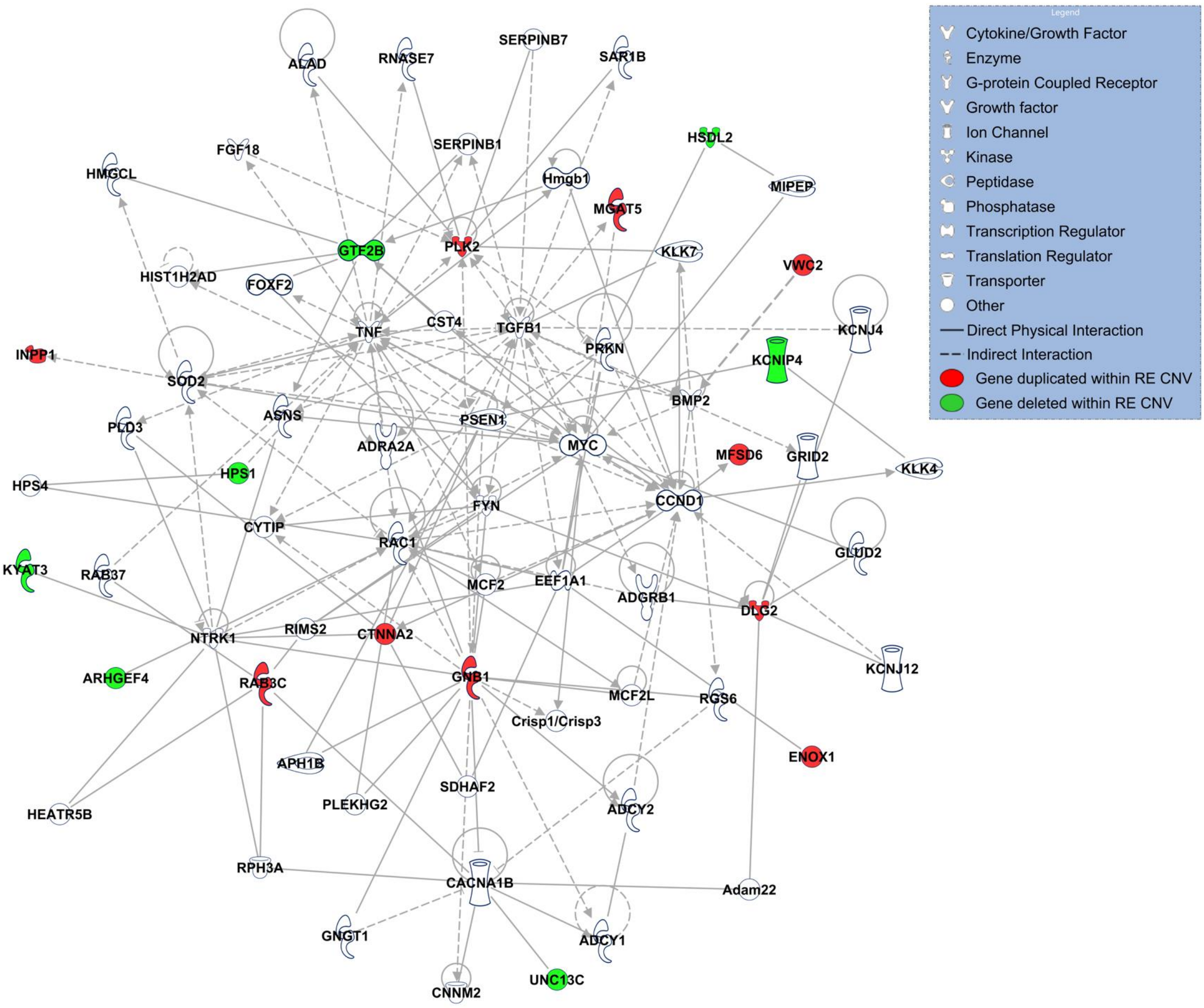

Supplementary Figure S3. Ingenuity Pathway Analysis of genes disrupted by CNV from European RE cases with high brain expression, creating a network of up to 70 genes. White symbols are genes added by IPA during network generation due to direct physical, or indirect (e.g. via activation) interactions with the input list.

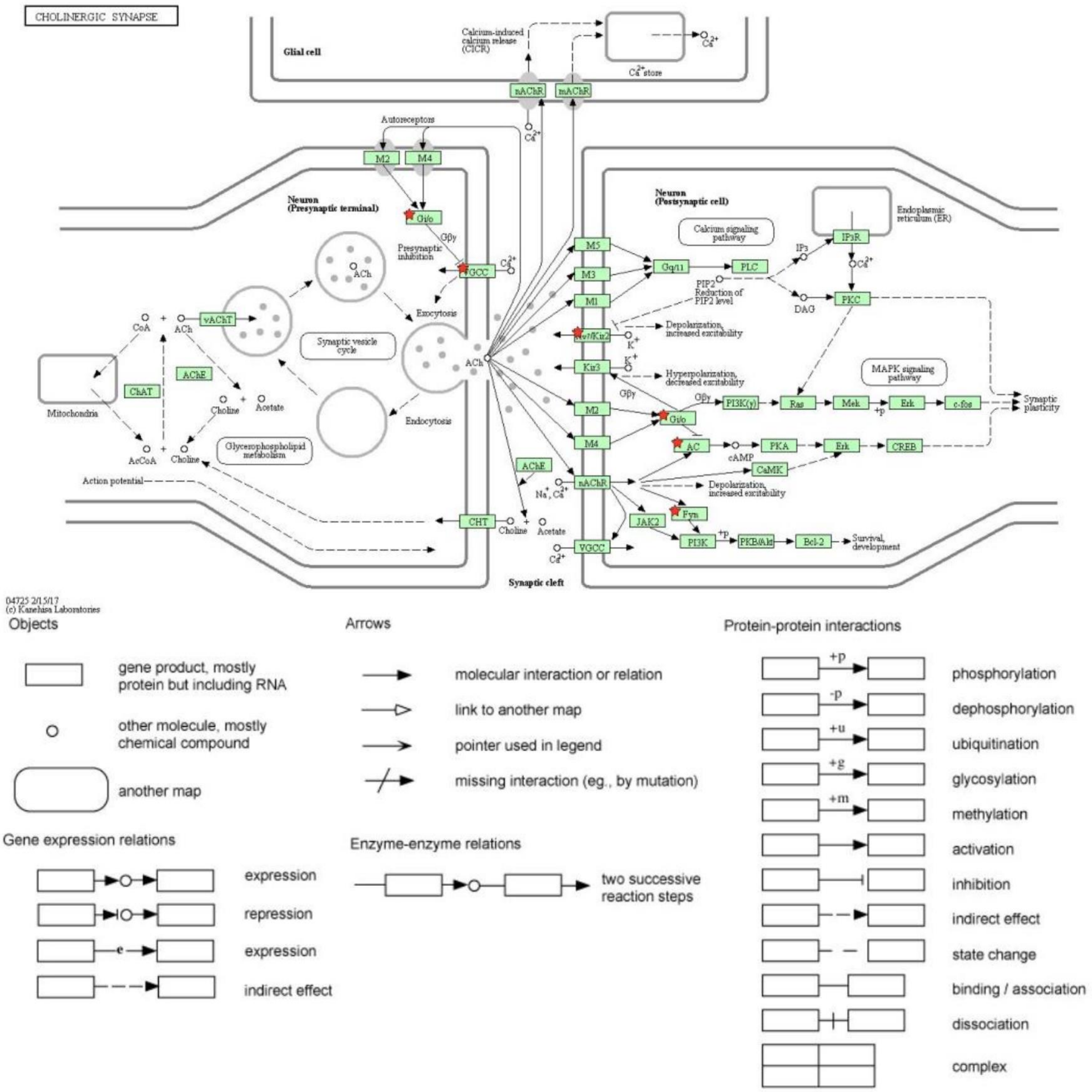

Supplementary Figure S4. KEGG pathway of cholinergic synapse from http://www.kegg.jp/. Red stars indicate genes that are from the IPA in Supplementary Figure 2. Enrichment analysis carried out within Enrichr http://amp.pharm.mssm.edu/Enrichr/ .

Supplementary Table S1

Cohort	Sample Source	Original Sample (N)	Family Members (N)	Cases (N)	Controls (N)
RE	UK	537	412	125	-
NL4	The Netherlands	800	-	-	800

The original Dutch cohort included 2,262 controls (dataset NL4)[1]. 800 controls from this dataset were randomly selected and used in the present study.

Supplementary Table S2

Cohort	Individuals after QC			SNPs	
	Samples (N)	Cases (N)	Controls (N)	Before QC (N)	After QC (N)
RE	76	76	-	538,448	227,632
NL4	795	-	795	719,665	612,666

After Quality Control, a total of 222,612 SNPs overlapped between the two datasets. Only affected individuals from the RE dataset were included in the QC to determine the population structure.

Supplementary Table S3

Case and Gene ID/hotspot	References
S218, *KLHL17*	2 3
SFR, 1q21.1	4 5
1050-301, *ARHGEF4*	6
RK044, *KCTD7*	7
RK011, *GRIN2A*	8-10
1012-301, 16p13.11	11-13
7083-301, *ARHGEF15*	14
All with Xp22.31 CNV	2 10 15-21
7007-301, *PTGER3*	22
RK029, *ASH1L*	23
1053-301, *CTNNA2*	24
S218, *ERBB4*	25 26
7037-301, *KCNIP4*	27
S52, *GRID2*	28
1029-301, *PLK2*	29 30
7007-301, *ARFGEF1*	31 32
SSM, *CTNNA3*	33 34
S38, *NRG3*	35
1041-301, *DLG2*	36
7021-301, *GRIP1*	37 38
1027-301, *UNC13*	39
SMJ, *SMAD3*	40
7034-301, *ASIC2*	41
NVH, *PTPRT*	42
1027-301, *SLITRK2*	43

Additional references for genes and regions disrupted by RE CNV and detailed in Tables 1 and 2 in the main text.

Term	Overlap	P-value	Adjusted P-value	Z-score	Combined Score	Genes
Cholinergic synapse_Homo sapiens_hsa04725	8/111	5.55E-09	9.60E-07	-2.06	39.18	GNGT1;KCNJ4;KCNJ12;GNB1;CACNA1B;ADCY2;FYN;ADCY1
Pathways in cancer_Homo sapiens_hsa05200	12/397	1.41E-08	1.22E-06	-2.08	37.65	NTRK1;GNGT1;BMP2;TGFB1;CCND1;MYC;FGF18;GNB1;ADCY2;ADCY1;CTNNA2;RAC1
GABAergic synapse_Homo sapiens_hsa04727	5/88	1.52E-05	6.20E-04	-1.80	19.92	GNGT1;GNB1;CACNA1B;ADCY2;ADCY1
Morphine addiction_Homo sapiens_hsa05032	5/91	1.79E-05	6.20E-04	-1.75	19.07	GNGT1;GNB1;CACNA1B;ADCY2;ADCY1
Hippo signaling pathway_Homo sapiens_hsa04390	6/153	1.73E-05	6.20E-04	-1.66	18.25	BMP2;TGFB1;DLG2;CCND1;MYC;CTNNA2
MAPK signaling pathway_Homo sapiens_hsa04010	7/255	3.35E-05	8.27E-04	-1.85	19.01	NTRK1;TGFB1;MYC;FGF18;CACNA1B;RAC1;TNF
Retrograde endocannabinoid signaling_Homo sapiens_hsa04723	5/101	2.97E-05	8.27E-04	-1.80	18.74	GNGT1;GNB1;CACNA1B;ADCY2;ADCY1
Colorectal cancer_Homo sapiens_hsa05210	4/62	6.96E-05	1.51E-03	-1.73	16.55	TGFB1;CCND1;MYC;RAC1
Thyroid cancer_Homo sapiens_hsa05216	3/29	1.47E-04	2.82E-03	-1.36	11.98	NTRK1;CCND1;MYC
Oxytocin signaling pathway_Homo sapiens_hsa04921	5/158	2.47E-04	3.88E-03	-1.71	14.20	KCNJ4;CCND1;KCNJ12;ADCY2;ADCY1
TGF-beta signaling pathway_Homo sapiens_hsa04350	4/84	2.27E-04	3.88E-03	-1.60	13.44	BMP2;TGFB1;MYC;TNF
HTLV-I infection_Homo sapiens_hsa05166	6/258	3.09E-04	4.11E-03	-1.64	13.28	TGFB1;CCND1;MYC;ADCY2;ADCY1;TNF
Dilated cardiomyopathy_Homo sapiens_hsa05414	4/90	2.96E-04	4.11E-03	-1.56	12.71	TGFB1;ADCY2;ADCY1;TNF
Circadian entrainment_Homo sapiens_hsa04713	4/95	3.64E-04	4.50E-03	-1.57	12.40	GNGT1;GNB1;ADCY2;ADCY1
AGE-RAGE signaling pathway in diabetic complications_Homo sapiens_hsa04933	4/101	4.59E-04	4.97E-03	-1.63	12.55	TGFB1;CCND1;RAC1;TNF
Amoebiasis_Homo sapiens_hsa05146	4/100	4.42E-04	4.97E-03	-1.48	11.44	SERPINB1;TGFB1;ADCY1;TNF
Chemokine signaling pathway_Homo sapiens_hsa04062	5/187	5.35E-04	5.45E-03	-1.51	11.40	GNGT1;GNB1;ADCY2;ADCY1;RAC1

Supplementary Table S4: Output table from Enricher of enriched KEGG pathways from IPA analysis of CNV-disrupted genes with high brain expression in European cases.

Term	Overlap	P-value	Adjusted P-value	Z-score	Combined Score	Genes
GTPase activity, coupled (GO:0061745)	6/151	1.6E-05	3.8E-03	-2.57	28.38	EEF1A1;GNGT1;RAB3C;SAR1B;GNB1;RAC1
GTPase activity (GO:0003924)	6/157	2.0E-05	3.8E-03	-2.61	28.23	EEF1A1;GNGT1;RAB3C;SAR1B;GNB1;RAC1
GTPase motor activity (GO:0061791)	6/169	3.0E-05	3.8E-03	-2.57	26.79	EEF1A1;GNGT1;RAB3C;SAR1B;GNB1;RAC1
Rho guanyl-nucleotide exchange factor activity (GO:0005089)	4/52	3.5E-05	3.8E-03	-1.96	20.11	PLEKHG2;MCF2L;MCF2;ARHGEF4
Rac guanyl-nucleotide exchange factor activity (GO:0030676)	4/56	4.7E-05	4.1E-03	-2.05	20.49	PLEKHG2;MCF2L;MCF2;ARHGEF4
PDZ domain binding (GO:0030165)	4/62	7.0E-05	5.0E-03	-2.04	19.57	PRKN;KCNJ4;GRID2;PSEN1

Supplementary Table S5: Output table from Enricher of enriched GO Molecular Functions from IPA analysis of CNV-disrupted genes with high brain expression in European cases.

Term	Overlap	P-value	Adjusted P-value	Z-score	Combined Score	Genes
positive regulation of apoptotic process (GO:0043065)	12/374	7.28E-09	9.95E-06	-4.87	91.24	NTRK1;BMP2;TGFB1;APH1B;PLEKHG2;MCF2;MCF2L;ARHGEF4;FYN;RAC1;HMGB1;TNF
canonical Wnt signaling pathway involved in positive regulation of apoptotic process (GO:0044337)	11/279	3.90E-09	9.95E-06	-4.36	84.39	BMP2;APH1B;PLEKHG2;MYC;MCF2;MCF2L;ARHGEF4;RAC1;PSEN1;HMGB1;TNF
positive regulation of cysteine-type endopeptidase activity involved in apoptotic process (GO:0043280)	10/258	2.48E-08	2.26E-05	-4.13	72.31	BMP2;APH1B;PLEKHG2;MYC;MCF2;MCF2L;ARHGEF4;RAC1;HMGB1;TNF
positive regulation of neuron apoptotic process (GO:0043525)	10/270	3.81E-08	2.60E-05	-4.25	72.68	BMP2;GRID2;APH1B;PLEKHG2;MCF2;MCF2L;ARHGEF4;RAC1;HMGB1;TNF
positive regulation of leukocyte apoptotic process (GO:2000108)	9/234	1.40E-07	2.64E-05	-3.97	62.61	BMP2;APH1B;PLEKHG2;MCF2;MCF2L;ARHGEF4;RAC1;HMGB1;TNF
positive regulation of fat cell apoptotic process (GO:1904651)	9/234	1.40E-07	2.64E-05	-3.97	62.60	BMP2;APH1B;PLEKHG2;MCF2;MCF2L;ARHGEF4;RAC1;HMGB1;TNF
positive regulation of myofibroblast cell apoptotic process (GO:1904522)	9/234	1.40E-07	2.64E-05	-3.96	62.58	BMP2;APH1B;PLEKHG2;MCF2;MCF2L;ARHGEF4;RAC1;HMGB1;TNF
positive regulation of muscle cell apoptotic process (GO:0010661)	9/234	1.40E-07	2.64E-05	-3.96	62.50	BMP2;APH1B;PLEKHG2;MCF2;MCF2L;ARHGEF4;RAC1;HMGB1;TNF
positive regulation of epithelial cell apoptotic process (GO:1904037)	9/234	1.40E-07	2.64E-05	-3.96	62.50	BMP2;APH1B;PLEKHG2;MCF2;MCF2L;ARHGEF4;RAC1;HMGB1;TNF
positive regulation of myeloid cell apoptotic process (GO:0033034)	9/235	1.45E-07	2.64E-05	-3.96	62.42	BMP2;APH1B;PLEKHG2;MCF2;MCF2L;ARHGEF4;RAC1;HMGB1;TNF
positive regulation of glial cell apoptotic process (GO:0034352)	9/234	1.40E-07	2.64E-05	-3.95	62.42	BMP2;APH1B;PLEKHG2;MCF2;MCF2L;ARHGEF4;RAC1;HMGB1;TNF
positive regulation of mesenchymal cell apoptotic process (GO:2001055)	9/234	1.40E-07	2.64E-05	-3.95	62.41	BMP2;APH1B;PLEKHG2;MCF2;MCF2L;ARHGEF4;RAC1;HMGB1;TNF
positive regulation of apoptotic process involved in development (GO:1904747)	9/234	1.40E-07	2.64E-05	-3.95	62.37	BMP2;APH1B;PLEKHG2;MCF2;MCF2L;ARHGEF4;RAC1;HMGB1;TNF
positive regulation of apoptotic process in other organism (GO:0044533)	9/234	1.40E-07	2.64E-05	-3.95	62.34	BMP2;APH1B;PLEKHG2;MCF2;MCF2L;ARHGEF4;RAC1;HMGB1;TNF
positive regulation of compound eye retinal cell apoptotic process (GO:1901694)	9/234	1.40E-07	2.64E-05	-3.95	62.31	BMP2;APH1B;PLEKHG2;MCF2;MCF2L;ARHGEF4;RAC1;HMGB1;TNF
positive regulation of execution phase of apoptosis (GO:1900119)	9/241	1.79E-07	2.72E-05	-4.03	62.62	BMP2;APH1B;PLEKHG2;MCF2;MCF2L;ARHGEF4;RAC1;HMGB1;TNF
positive regulation of anoikis (GO:2000210)	9/240	1.73E-07	2.72E-05	-3.98	62.01	BMP2;APH1B;PLEKHG2;MCF2;MCF2L;ARHGEF4;RAC1;HMGB1;TNF
positive regulation of fibroblast apoptotic process (GO:2000271)	9/238	1.61E-07	2.72E-05	-3.94	61.69	BMP2;APH1B;PLEKHG2;MCF2;MCF2L;ARHGEF4;RAC1;HMGB1;TNF
positive regulation of apoptotic signaling pathway (GO:2001235)	9/248	2.29E-07	3.29E-05	-4.09	62.51	BMP2;APH1B;PLEKHG2;MCF2;MCF2L;ARHGEF4;RAC1;HMGB1;TNF
cellular response to forskolin (GO:1904322)	4/19	5.43E-07	7.41E-05	-3.42	49.30	TGFB1;ADCY2;ADCY1;TNF
Notch receptor processing, ligand-dependent (GO:0035333)	5/59	2.11E-06	2.74E-04	-2.57	33.62	BMP2;TGFB1;APH1B;MYC;PSEN1
positive regulation of I-kappaB phosphorylation (GO:1903721)	8/252	3.05E-06	3.47E-04	-4.03	51.22	PRKN;NTRK1;BMP2;TGFB1;CCND1;PLK2;RAC1;TNF
induction by virus of host protein phosphorylation (GO:0039614)	6/113	3.02E-06	3.47E-04	-2.90	36.80	NTRK1;BMP2;TGFB1;CCND1;RAC1;TNF
positive regulation of inhibitory G-protein coupled receptor phosphorylation (GO:1904325)	6/113	3.02E-06	3.47E-04	-2.89	36.77	NTRK1;BMP2;TGFB1;CCND1;RAC1;TNF
positive regulation of MAP kinase activity (GO:0043406)	6/118	3.89E-06	4.25E-04	-3.06	38.16	BMP2;TGFB1;FGF18;HMGB1;TNF;ADRA2A
positive regulation of histone phosphorylation (GO:0033129)	6/120	4.29E-06	4.50E-04	-2.88	35.54	NTRK1;BMP2;TGFB1;CCND1;RAC1;TNF
positive regulation of phosphorylation of RNA polymerase II C-terminal domain (GO:1901409)	6/122	4.72E-06	4.77E-04	-2.95	36.17	NTRK1;BMP2;TGFB1;CCND1;RAC1;TNF
positive regulation of MAPK cascade (GO:0043410)	7/194	5.71E-06	5.57E-04	-3.41	41.19	NTRK1;BMP2;TGFB1;CCND1;RAC1;HMGB1;TNF
positive regulation of protein autophosphorylation (GO:0031954)	6/128	6.22E-06	5.86E-04	-3.05	36.54	NTRK1;BMP2;TGFB1;CCND1;RAC1;TNF
positive regulation of peptidyl-threonine phosphorylation (GO:0010800)	6/131	7.11E-06	6.48E-04	-3.10	36.73	NTRK1;BMP2;TGFB1;CCND1;RAC1;TNF
activation of MAPKK activity (GO:0000186)	5/86	1.36E-05	1.20E-03	-3.03	33.92	NTRK1;TGFB1;FGF18;TNF;ADRA2A
positive regulation of ERK1 and ERK2 cascade via TNFSF11-mediated signaling (GO:0071848)	6/151	1.60E-05	1.33E-03	-3.28	36.26	NTRK1;BMP2;TGFB1;FGF18;HMGB1;TNF
positive regulation of protein phosphorylation (GO:0001934)	6/151	1.60E-05	1.33E-03	-3.26	36.03	NTRK1;BMP2;TGFB1;CCND1;RAC1;TNF
positive regulation of pathway-restricted SMAD protein phosphorylation (GO:0010862)	6/155	1.86E-05	1.49E-03	-3.21	34.94	NTRK1;BMP2;TGFB1;CCND1;RAC1;TNF
positive regulation of peptidyl-serine phosphorylation (GO:0033138)	6/160	2.22E-05	1.74E-03	-3.46	37.11	NTRK1;BMP2;TGFB1;CCND1;RAC1;TNF
positive regulation of peptidyl-tyrosine phosphorylation (GO:0050731)	6/169	3.03E-05	2.30E-03	-3.40	35.41	NTRK1;BMP2;TGFB1;CCND1;RAC1;TNF
cellular response to catecholamine stimulus (GO:0071870)	3/18	3.37E-05	2.49E-03	-2.96	30.49	TGFB1;GNB1;TNF
positive regulation of protein kinase activity (GO:0045860)	6/176	3.80E-05	2.66E-03	-3.40	34.62	NTRK1;BMP2;TGFB1;CCND1;RAC1;TNF
positive regulation of JUN kinase activity (GO:0043507)	5/106	3.75E-05	2.66E-03	-2.87	29.22	TGFB1;FGF18;HMGB1;TNF;ADRA2A
cAMP-mediated signaling (GO:0019933)	3/23	7.22E-05	4.93E-03	-2.94	28.04	RIMS2;ADCY2;ADCY1
negative regulation of neuron apoptotic process (GO:0043524)	8/399	8.31E-05	5.40E-03	-5.02	47.19	NTRK1;PRKN;GRID2;MYC;PLK2;ASNS;PSEN1;SOD2

Supplementary Table S6: Output table from Enricher of enriched GO Biological Processes from IPA analysis of CNV-disrupted genes with high brain expression in European cases.

[illegible]

Term	Overlap	P-value	Adjusted P-value	Z-score	Combined Score	Genes
**mRNA binding (GO:0003729)**	18/1230	1.52E-07	6.78E-06	-3.87	60.74	YWHAE;EIF4A2;LRRC59;SF3B2;RBFOX3;LARP4B;APEH;C14ORF93;GTPBP4;AKAP1;MOV10;S100A16;KYAT3;LUC7L3;RP9;METTL16;SRSF7;FAM50A
**RNA binding (GO:0003723)**	17/1199	5.68E-07	6.78E-06	-3.68	52.90	YWHAE;EIF4A2;LRRC59;SF3B2;LARP4B;APEH;C14ORF93;GTPBP4;AKAP1;MOV10;S100A16;KYAT3;LUC7L3;RP9;METTL16;SRSF7;FAM50A
**base pairing with RNA (GO:0000498)**	17/1199	5.68E-07	6.78E-06	-3.67	52.81	YWHAE;EIF4A2;LRRC59;SF3B2;LARP4B;APEH;C14ORF93;GTPBP4;AKAP1;MOV10;S100A16;KYAT3;LUC7L3;RP9;METTL16;SRSF7;FAM50A
**N6-methyladenosine-containing RNA binding (GO:1990247)**	17/1199	5.68E-07	6.78E-06	-3.67	52.73	YWHAE;EIF4A2;LRRC59;SF3B2;LARP4B;APEH;C14ORF93;GTPBP4;AKAP1;MOV10;S100A16;KYAT3;LUC7L3;RP9;METTL16;SRSF7;FAM50A
**pre-mRNA binding (GO:0036002)**	17/1200	5.75E-07	6.78E-06	-3.67	52.68	YWHAE;EIF4A2;LRRC59;SF3B2;LARP4B;APEH;C14ORF93;GTPBP4;AKAP1;MOV10;S100A16;KYAT3;LUC7L3;RP9;METTL16;SRSF7;FAM50A
**7S RNA binding (GO:0008312)**	17/1200	5.75E-07	6.78E-06	-3.66	52.58	YWHAE;EIF4A2;LRRC59;SF3B2;LARP4B;APEH;C14ORF93;GTPBP4;AKAP1;MOV10;S100A16;KYAT3;LUC7L3;RP9;METTL16;SRSF7;FAM50A
**misfolded RNA binding (GO:0034336)**	17/1199	5.68E-07	6.78E-06	-3.66	52.56	YWHAE;EIF4A2;LRRC59;SF3B2;LARP4B;APEH;C14ORF93;GTPBP4;AKAP1;MOV10;S100A16;KYAT3;LUC7L3;RP9;METTL16;SRSF7;FAM50A
**G-quadruplex RNA binding (GO:0002151)**	17/1201	5.82E-07	6.78E-06	-3.66	52.53	YWHAE;EIF4A2;LRRC59;SF3B2;LARP4B;APEH;C14ORF93;GTPBP4;AKAP1;MOV10;S100A16;KYAT3;LUC7L3;RP9;METTL16;SRSF7;FAM50A
**histone pre-mRNA DCP binding (GO:0071208)**	17/1200	5.75E-07	6.78E-06	-3.65	52.50	YWHAE;EIF4A2;LRRC59;SF3B2;LARP4B;APEH;C14ORF93;GTPBP4;AKAP1;MOV10;S100A16;KYAT3;LUC7L3;RP9;METTL16;SRSF7;FAM50A
**alpha-aminoacyl-tRNA binding (GO:1904678)**	17/1199	5.68E-07	6.78E-06	-3.65	52.50	YWHAE;EIF4A2;LRRC59;SF3B2;LARP4B;APEH;C14ORF93;GTPBP4;AKAP1;MOV10;S100A16;KYAT3;LUC7L3;RP9;METTL16;SRSF7;FAM50A
**snRNA binding (GO:0017069)**	17/1202	5.89E-07	6.78E-06	-3.66	52.48	YWHAE;EIF4A2;LRRC59;SF3B2;LARP4B;APEH;C14ORF93;GTPBP4;AKAP1;MOV10;S100A16;KYAT3;LUC7L3;RP9;METTL16;SRSF7;FAM50A
**AU-rich element binding (GO:0017091)**	17/1203	5.95E-07	6.78E-06	-3.66	52.42	YWHAE;EIF4A2;LRRC59;SF3B2;LARP4B;APEH;C14ORF93;GTPBP4;AKAP1;MOV10;S100A16;KYAT3;LUC7L3;RP9;METTL16;SRSF7;FAM50A
**miRNA binding (GO:0035198)**	17/1203	5.95E-07	6.78E-06	-3.65	52.38	YWHAE;EIF4A2;LRRC59;SF3B2;LARP4B;APEH;C14ORF93;GTPBP4;AKAP1;MOV10;S100A16;KYAT3;LUC7L3;RP9;METTL16;SRSF7;FAM50A
**21U-RNA binding (GO:0034583)**	17/1199	5.68E-07	6.78E-06	-3.64	52.37	YWHAE;EIF4A2;LRRC59;SF3B2;LARP4B;APEH;C14ORF93;GTPBP4;AKAP1;MOV10;S100A16;KYAT3;LUC7L3;RP9;METTL16;SRSF7;FAM50A
**pre-miRNA binding (GO:0070883)**	17/1201	5.82E-07	6.78E-06	-3.65	52.37	YWHAE;EIF4A2;LRRC59;SF3B2;LARP4B;APEH;C14ORF93;GTPBP4;AKAP1;MOV10;S100A16;KYAT3;LUC7L3;RP9;METTL16;SRSF7;FAM50A
**telomeric repeat-containing RNA binding (GO:0061752)**	17/1200	5.75E-07	6.78E-06	-3.64	52.36	YWHAE;EIF4A2;LRRC59;SF3B2;LARP4B;APEH;C14ORF93;GTPBP4;AKAP1;MOV10;S100A16;KYAT3;LUC7L3;RP9;METTL16;SRSF7;FAM50A
**RNA stem-loop binding (GO:0035613)**	17/1200	5.75E-07	6.78E-06	-3.64	52.30	YWHAE;EIF4A2;LRRC59;SF3B2;LARP4B;APEH;C14ORF93;GTPBP4;AKAP1;MOV10;S100A16;KYAT3;LUC7L3;RP9;METTL16;SRSF7;FAM50A
**GU repeat RNA binding (GO:1990605)**	17/1199	5.68E-07	6.78E-06	-3.63	52.26	YWHAE;EIF4A2;LRRC59;SF3B2;LARP4B;APEH;C14ORF93;GTPBP4;AKAP1;MOV10;S100A16;KYAT3;LUC7L3;RP9;METTL16;SRSF7;FAM50A
**RNA cap binding (GO:0000339)**	17/1201	5.82E-07	6.78E-06	-3.64	52.24	YWHAE;EIF4A2;LRRC59;SF3B2;LARP4B;APEH;C14ORF93;GTPBP4;AKAP1;MOV10;S100A16;KYAT3;LUC7L3;RP9;METTL16;SRSF7;FAM50A
**piRNA binding (GO:0034584)**	17/1202	5.89E-07	6.78E-06	-3.64	52.22	YWHAE;EIF4A2;LRRC59;SF3B2;LARP4B;APEH;C14ORF93;GTPBP4;AKAP1;MOV10;S100A16;KYAT3;LUC7L3;RP9;METTL16;SRSF7;FAM50A
**BRE binding (GO:0042835)**	17/1200	5.75E-07	6.78E-06	-3.63	52.20	YWHAE;EIF4A2;LRRC59;SF3B2;LARP4B;APEH;C14ORF93;GTPBP4;AKAP1;MOV10;S100A16;KYAT3;LUC7L3;RP9;METTL16;SRSF7;FAM50A
**ribonuclease P RNA binding (GO:0033204)**	17/1199	5.68E-07	6.78E-06	-3.63	52.20	YWHAE;EIF4A2;LRRC59;SF3B2;LARP4B;APEH;C14ORF93;GTPBP4;AKAP1;MOV10;S100A16;KYAT3;LUC7L3;RP9;METTL16;SRSF7;FAM50A
**telomerase RNA binding (GO:0070034)**	17/1205	6.09E-07	6.78E-06	-3.64	52.15	YWHAE;EIF4A2;LRRC59;SF3B2;LARP4B;APEH;C14ORF93;GTPBP4;AKAP1;MOV10;S100A16;KYAT3;LUC7L3;RP9;METTL16;SRSF7;FAM50A
**snoRNA binding (GO:0030515)**	17/1206	6.16E-07	6.78E-06	-3.65	52.14	YWHAE;EIF4A2;LRRC59;SF3B2;LARP4B;APEH;C14ORF93;GTPBP4;AKAP1;MOV10;S100A16;KYAT3;LUC7L3;RP9;METTL16;SRSF7;FAM50A
**primary miRNA binding (GO:0070878)**	17/1204	6.02E-07	6.78E-06	-3.64	52.13	YWHAE;EIF4A2;LRRC59;SF3B2;LARP4B;APEH;C14ORF93;GTPBP4;AKAP1;MOV10;S100A16;KYAT3;LUC7L3;RP9;METTL16;SRSF7;FAM50A
**regulatory region RNA binding (GO:0001069)**	17/1199	5.68E-07	6.78E-06	-3.62	52.10	YWHAE;EIF4A2;LRRC59;SF3B2;LARP4B;APEH;C14ORF93;GTPBP4;AKAP1;MOV10;S100A16;KYAT3;LUC7L3;RP9;METTL16;SRSF7;FAM50A
**rRNA binding (GO:0019843)**	17/1205	6.09E-07	6.78E-06	-3.64	52.04	YWHAE;EIF4A2;LRRC59;SF3B2;LARP4B;APEH;C14ORF93;GTPBP4;AKAP1;MOV10;S100A16;KYAT3;LUC7L3;RP9;METTL16;SRSF7;FAM50A
**siRNA binding (GO:0035197)**	17/1203	5.95E-07	6.78E-06	-3.63	52.04	YWHAE;EIF4A2;LRRC59;SF3B2;LARP4B;APEH;C14ORF93;GTPBP4;AKAP1;MOV10;S100A16;KYAT3;LUC7L3;RP9;METTL16;SRSF7;FAM50A
**translation factor activity, RNA binding (GO:0008135)**	17/1210	6.46E-07	6.86E-06	-3.65	52.06	YWHAE;EIF4A2;LRRC59;SF3B2;LARP4B;APEH;C14ORF93;GTPBP4;AKAP1;MOV10;S100A16;KYAT3;LUC7L3;RP9;METTL16;SRSF7;FAM50A
**single-stranded RNA binding (GO:0003727)**	17/1216	6.92E-07	7.10E-06	-3.66	51.88	YWHAE;EIF4A2;LRRC59;SF3B2;LARP4B;APEH;C14ORF93;GTPBP4;AKAP1;MOV10;S100A16;KYAT3;LUC7L3;RP9;METTL16;SRSF7;FAM50A
**tRNA binding (GO:0000049)**	17/1219	7.16E-07	7.11E-06	-3.65	51.62	YWHAE;EIF4A2;LRRC59;SF3B2;LARP4B;APEH;C14ORF93;GTPBP4;AKAP1;MOV10;S100A16;KYAT3;LUC7L3;RP9;METTL16;SRSF7;FAM50A
**double-stranded RNA binding (GO:0003725)**	17/1223	7.49E-07	7.21E-06	-3.62	51.04	YWHAE;EIF4A2;LRRC59;SF3B2;LARP4B;APEH;C14ORF93;GTPBP4;AKAP1;MOV10;S100A16;KYAT3;LUC7L3;RP9;METTL16;SRSF7;FAM50A
**death receptor binding (GO:0005123)**	2/14	1.07E-03	9.98E-03	-1.03	7.07	TMBIM1;TNFSF11

Supplementary Table S7: Output table from Enricher of enriched GO Molecular Functions from IPA analysis of CNV-disrupted genes with high brain expression in European controls.

REFERENCES

1. van Rheenen W, Shatunov A, Dekker AM, McLaughlin RL, Diekstra FP, Pulit SL, van der Spek RA, Vosa U, de Jong S, Robinson MR, Yang J, Fogh I, van Doormaal PT, Tazelaar GH, Koppers M, Blokhuis AM, Sproviero W, Jones AR, Kenna KP, van Eijk KR, Harschnitz O, Schellevis RD, Brands WJ, Medic J, Menelaou A, Vajda A, Ticozzi N, Lin K, Rogelj B, Vrabec K, Ravnik-Glavac M, Koritnik B, Zidar J, Leonardis L, Groselj LD, Millecamps S, Salachas F, Meininger V, de Carvalho M, Pinto S, Mora JS, Rojas-Garcia R, Polak M, Chandran S, Colville S, Swingler R, Morrison KE, Shaw PJ, Hardy J, Orrell RW, Pittman A, Sidle K, Fratta P, Malaspina A, Topp S, Petri S, Abdulla S, Drepper C, Sendtner M, Meyer T, Ophoff RA, Staats KA, Wiedau-Pazos M, Lomen-Hoerth C, Van Deerlin VM, Trojanowski JQ, Elman L, McCluskey L, Basak AN, Tunca C, Hamzeiy H, Parman Y, Meitinger T, Lichtner P, Radivojkov-Blagojevic M, Andres CR, Maurel C, Bensimon G, Landwehrmeyer B, Brice A, Payan CA, Saker-Delye S, Durr A, Wood NW, Tittmann L, Lieb W, Franke A, Rietschel M, Cichon S, Nothen MM, Amouyel P, Tzourio C, Dartigues JF, Uitterlinden AG, Rivadeneira F, Estrada K, Hofman A, Curtis C, Blauw HM, van der Kooi AJ, de Visser M, Goris A, Weber M, Shaw CE, Smith BN, Pansarasa O, Cereda C, Del Bo R, Comi GP, D'Alfonso S, Bertolin C, Soraru G, Mazzini L, Pensato V, Gellera C, Tiloca C, Ratti A, Calvo A, Moglia C, Brunetti M, Arcuti S, Capozzo R, Zecca C, Lunetta C, Penco S, Riva N, Padovani A, Filosto M, Muller B, Stuit RJ, Registry P, Group S, Registry S, Consortium FS, Consortium S, Group NS, Blair I, Zhang K, McCann EP, Fifita JA, Nicholson GA, Rowe DB, Pamphlett R, Kiernan MC, Grosskreutz J, Witte OW, Ringer T, Prell T, Stubendorff B, Kurth I, Hubner CA, Leigh PN, Casale F, Chio A, Beghi E, Pupillo E, Tortelli R, Logroscino G, Powell J, Ludolph AC, Weishaupt JH, Robberecht W, Van Damme P, Franke L, Pers TH, Brown RH, Glass JD, Landers JE, Hardiman O, Andersen PM, Corcia P, Vourc'h P, Silani V, Wray NR, Visscher PM, de Bakker PI, van Es MA, Pasterkamp RJ, Lewis CM, Breen G, Al-Chalabi A, van den Berg LH, Veldink JH. Genome-wide association analyses identify new risk variants and the genetic architecture of amyotrophic lateral sclerosis. Nat Genet 2016;48(9):1043-8. doi: 10.1038/ng.3622 [published Online First: 2016/07/28]
2. Addis L, Rosch RE, Valentin A, Makoff A, Robinson R, Everett KV, Nashef L, Pal DK. Analysis of rare copy number variation in absence epilepsies. Neurology Genetics 2016;2(2):e56. doi: 10.1212/NXG.0000000000000056
3. Paciorkowski AR, Thio LL, Rosenfeld JA, Gajecka M, Gurnett CA, Kulkarni S, Chung WK, Marsh ED, Gentile M, Reggin JD, Wheless JW, Balasubramanian S, Kumar R, Christian SL, Marini C, Guerrini R, Maltsev N, Shaffer LG, Dobyns WB. Copy number variants and infantile spasms: evidence for abnormalities in ventral forebrain development and pathways of synaptic function. Eur J Hum Genet 2011;19(12):1238-45. doi: ejhg2011121 [pii] 10.1038/ejhg.2011.121 [published Online First: 2011/06/23]
4. Mefford HC, Sharp AJ, Baker C, Itsara A, Jiang Z, Buysse K, Huang S, Maloney VK, Crolla JA, Baralle D, Collins A, Mercer C, Norga K, de Ravel T, Devriendt K, Bongers EM, de Leeuw N, Reardon W, Gimelli S, Bena F, Hennekam RC, Male A, Gaunt L, Clayton-Smith J, Simonic I, Park SM, Mehta SG, Nik-Zainal S, Woods CG, Firth HV, Parkin G, Fichera M, Reitano S, Lo Giudice M, Li KE, Casuga I, Broomer A, Conrad B, Schwerzmann M, Raber L, Gallati S, Striano P, Coppola A, Tolmie JL, Tobias ES, Lilley C, Armengol L, Spysschaert Y, Verloo P, De Coene A, Goossens L, Mortier G, Speleman F, van Binsbergen E, Nelen MR, Hochstenbach R, Poot M, Gallagher L, Gill M, McClellan J, King MC, Regan R, Skinner C, Stevenson RE, Antonarakis SE, Chen C, Estivill X, Menten B, Gimelli G, Gribble S, Schwartz S, Sutcliffe JS, Walsh T, Knight SJ, Sebat J, Romano C, Schwartz CE, Veltman JA, de Vries BB, Vermeesch JR, Barber JC, Willatt L, Tassabehji M, Eichler EE. Recurrent rearrangements of chromosome 1q21.1 and variable pediatric phenotypes. N Engl J Med 2008;359(16):1685-99. doi: NEJMoa0805384 [pii] 10.1056/NEJMoa0805384 [published Online First: 2008/09/12]
5. Rosenfeld JA, Traylor RN, Schaefer GB, McPherson EW, Ballif BC, Klopocki E, Mundlos S, Shaffer LG, Aylsworth AS. Proximal microdeletions and microduplications of 1q21.1 contribute to variable abnormal phenotypes. Eur J Hum Genet 2012;20(7):754-61. doi: ejhg20126 [pii] 10.1038/ejhg.2012.6 [published Online First: 2012/02/10]

6. Dharmadhikari AV, Kang SH, Szafranski P, Person RE, Sampath S, Prakash SK, Bader PI, Phillips JA, 3rd, Hannig V, Williams M, Vinson SS, Wilfong AA, Reimschisel TE, Craigen WJ, Patel A, Bi W, Lupski JR, Belmont J, Cheung SW, Stankiewicz P. Small rare recurrent deletions and reciprocal duplications in 2q21.1, including brain-specific ARHGEF4 and GPR148. Hum Mol Genet 2012;21(15):3345-55. doi: dds166 [pii] 10.1093/hmg/dds166 [published Online First: 2012/05/01]
7. Kousi M, Anttila V, Schulz A, Calafato S, Jakkula E, Riesch E, Myllykangas L, Kalimo H, Topcu M, Gokben S, Alehan F, Lemke JR, Alber M, Palotie A, Kopra O, Lehesjoki AE. Novel mutations consolidate KCTD7 as a progressive myoclonus epilepsy gene. J Med Genet 2012;49(6):391-9. doi: jmedgenet-2012-100859 [pii] 10.1136/jmedgenet-2012-100859 [published Online First: 2012/06/14]
8. Carvill GL, Regan BM, Yendle SC, O'Roak BJ, Lozovaya N, Bruneau N, Burnashev N, Khan A, Cook J, Geraghty E, Sadleir LG, Turner SJ, Tsai MH, Webster R, Ouvrier R, Damiano JA, Berkovic SF, Shendure J, Hildebrand MS, Szepetowski P, Scheffer IE, Mefford HC. GRIN2A mutations cause epilepsy-aphasia spectrum disorders. Nat Genet 2013;45(9):1073-6. doi: ng.2727 [pii] 10.1038/ng.2727 [published Online First: 2013/08/13]
9. Lemke JR, Lal D, Reinthaler EM, Steiner I, Nothnagel M, Alber M, Geider K, Laube B, Schwake M, Finsterwalder K, Franke A, Schilhabel M, Jahn JA, Muhle H, Boor R, Van Paesschen W, Caraballo R, Fejerman N, Weckhuysen S, De Jonghe P, Larsen J, Moller RS, Hjalgrim H, Addis L, Tang S, Hughes E, Pal DK, Veri K, Vaher U, Talvik T, Dimova P, Guerrero Lopez R, Serratosa JM, Linnankivi T, Lehesjoki AE, Ruf S, Wolff M, Buerki S, Wohlrab G, Kroell J, Datta AN, Fiedler B, Kurlemann G, Kluger G, Hahn A, Haberlandt DE, Kutzer C, Sperner J, Becker F, Weber YG, Feucht M, Steinbock H, Neophythou B, Ronen GM, Gruber-Sedlmayr U, Geldner J, Harvey RJ, Hoffmann P, Herms S, Altmuller J, Toliat MR, Thiele H, Nurnberg P, Wilhelm C, Stephani U, Helbig I, Lerche H, Zimprich F, Neubauer BA, Biskup S, von Spiczak S. Mutations in GRIN2A cause idiopathic focal epilepsy with rolandic spikes. Nat Genet 2013;45(9):1067-72. doi: ng.2728 [pii] 10.1038/ng.2728 [published Online First: 2013/08/13]
10. Lesca G, Rudolf G, Bruneau N, Lozovaya N, Labalme A, Boutry-Kryza N, Salmi M, Tsintsadze T, Addis L, Motte J, Wright S, Tsintsadze V, Michel A, Doummar D, Lascelles K, Strug L, Waters P, de Bellescize J, Vrielynck P, de Saint Martin A, Ville D, Ryvlin P, Arzimanoglou A, Hirsch E, Vincent A, Pal D, Burnashev N, Sanlaville D, Szepetowski P. GRIN2A mutations in acquired epileptic aphasia and related childhood focal epilepsies and encephalopathies with speech and language dysfunction. Nat Genet 2013;45(9):1061-6. doi: ng.2726 [pii] 10.1038/ng.2726 [published Online First: 2013/08/13]
11. de Kovel CG, Trucks H, Helbig I, Mefford HC, Baker C, Leu C, Kluck C, Muhle H, von Spiczak S, Ostertag P, Obermeier T, Kleefuss-Lie AA, Hallmann K, Steffens M, Gaus V, Klein KM, Hamer HM, Rosenow F, Brilstra EH, Trenite DK, Swinkels ME, Weber YG, Unterberger I, Zimprich F, Urak L, Feucht M, Fuchs K, Moller RS, Hjalgrim H, De Jonghe P, Suls A, Ruckert IM, Wichmann HE, Franke A, Schreiber S, Nurnberg P, Elger CE, Lerche H, Stephani U, Koeleman BP, Lindhout D, Eichler EE, Sander T. Recurrent microdeletions at 15q11.2 and 16p13.11 predispose to idiopathic generalized epilepsies. Brain 2010;133(Pt 1):23-32. doi: awp262 [pii] 10.1093/brain/awp262 [published Online First: 2009/10/22]
12. Heinzen EL, Radtke RA, Urban TJ, Cavalleri GL, Depondt C, Need AC, Walley NM, Nicoletti P, Ge D, Catarino CB, Duncan JS, Kasperaviciute D, Tate SK, Caboclo LO, Sander JW, Clayton L, Linney KN, Shianna KV, Gumbs CE, Smith J, Cronin KD, Maia JM, Doherty CP, Pandolfo M, Leppert D, Middleton LT, Gibson RA, Johnson MR, Matthews PM, Hosford D, Kalviainen R, Eriksson K, Kantanen AM, Dorn T, Hansen J, Kramer G, Steinhoff BJ, Wieser HG, Zumsteg D, Ortega M, Wood NW, Huxley-Jones J, Mikati M, Gallentine WB, Husain AM, Buckley PG, Stallings RL, Podgoreanu MV, Delanty N, Sisodiya SM, Goldstein DB. Rare deletions at 16p13.11 predispose to a diverse spectrum of sporadic epilepsy syndromes. Am J Hum Genet 2010;86(5):707-18. doi: S0002-9297(10)00163-1 [pii] 10.1016/j.ajhg.2010.03.018 [published Online First: 2010/04/20]
13. Mefford HC, Muhle H, Ostertag P, von Spiczak S, Buysse K, Baker C, Franke A, Malafosse A, Genton P, Thomas P, Gurnett CA, Schreiber S, Bassuk AG, Guipponi M, Stephani U, Helbig I, Eichler EE. Genome-wide copy number variation in epilepsy: novel susceptibility loci in idiopathic generalized and focal epilepsies. PLoS Genet 2010;6(5):e1000962. doi: 10.1371/journal.pgen.1000962 [published Online First: 2010/05/27]

14. Veeramah KR, Johnstone L, Karafet TM, Wolf D, Sprissler R, Salogiannis J, Barth-Maron A, Greenberg ME, Stuhlmann T, Weinert S, Jentsch TJ, Pazzi M, Restifo LL, Talwar D, Erickson RP, Hammer MF. Exome sequencing reveals new causal mutations in children with epileptic encephalopathies. Epilepsia 2013;54(7):1270-81. doi: 10.1111/epi.12201 [published Online First: 2013/05/08]
15. Olson H, Shen Y, Avallone J, Sheidley BR, Pinsky R, Bergin AM, Berry GT, Duffy FH, Eksioglu Y, Harris DJ, Hisama FM, Ho E, Irons M, Jacobsen CM, James P, Kothare S, Khwaja O, Lipton J, Loddenkemper T, Markowitz J, Maski K, Megerian JT, Neilan E, Raffalli PC, Robbins M, Roberts A, Roe E, Rollins C, Sahin M, Sarco D, Schonwald A, Smith SE, Soul J, Stoler JM, Takeoka M, Tan WH, Torres AR, Tsai P, Urion DK, Weissman L, Wolff R, Wu BL, Miller DT, Poduri A. Copy number variation plays an important role in clinical epilepsy. Ann Neurol 2014;75(6):943-58. doi: 10.1002/ana.24178 [published Online First: 2014/05/09]
16. Reinthaler EM, Lal D, Lebon S, Hildebrand MS, Dahl HH, Regan BM, Feucht M, Steinbock H, Neophytou B, Ronen GM, Roche L, Gruber-Sedlmayr U, Geldner J, Haberlandt E, Hoffmann P, Herms S, Gieger C, Waldenberger M, Franke A, Wittig M, Schoch S, Becker AJ, Hahn A, Mannik K, Toliat MR, Winterer G, Lerche H, Nurnberg P, Mefford H, Scheffer IE, Berkovic SF, Beckmann JS, Sander T, Jacquemont S, Reymond A, Zimprich F, Neubauer BA. 16p11.2 600 kb Duplications confer risk for typical and atypical Rolandic epilepsy. Hum Mol Genet 2014;23(22):6069-80. doi: ddu306 [pii] 10.1093/hmg/ddu306 [published Online First: 2014/06/19]
17. Shaw-Smith C, Redon R, Rickman L, Rio M, Willatt L, Fiegler H, Firth H, Sanlaville D, Winter R, Colleaux L, Bobrow M, Carter NP. Microarray based comparative genomic hybridisation (array-CGH) detects submicroscopic chromosomal deletions and duplications in patients with learning disability/mental retardation and dysmorphic features. J Med Genet 2004;41(4):241-8. [published Online First: 2004/04/03]
18. Esplin ED, Li B, Slavotinek A, Novelli A, Battaglia A, Clark R, Curry C, Hudgins L. Nine patients with Xp22.31 microduplication, cognitive deficits, seizures, and talipes anomalies. Am J Med Genet A 2014;164A(8):2097-103. doi: 10.1002/ajmg.a.36598 [published Online First: 2014/05/08]
19. Li F, Shen Y, Kohler U, Sharkey FH, Menon D, Coulleaux L, Malan V, Rio M, McMullan DJ, Cox H, Fagan KA, Gaunt L, Metcalfe K, Heinrich U, Hislop G, Maye U, Sutcliffe M, Wu BL, Thiel BD, Mulchandani S, Conlin LK, Spinner NB, Murphy KM, Batista DA. Interstitial microduplication of Xp22.31: Causative of intellectual disability or benign copy number variant? Eur J Med Genet 2010;53(2):93-9. doi: S1769-7212(10)00007-8 [pii] 10.1016/j.ejmg.2010.01.004 [published Online First: 2010/02/06]
20. Gohlke BC, Haug K, Fukami M, Friedl W, Noeker M, Rappold GA, Haverkamp F. Interstitial deletion in Xp22.3 is associated with X linked ichthyosis, mental retardation, and epilepsy. J Med Genet 2000;37(8):600-2. [published Online First: 2000/08/03]
21. Tyson C, Harvard C, Locker R, Friedman JM, Langlois S, Lewis ME, Van Allen M, Somerville M, Arbour L, Clarke L, McGilivray B, Yong SL, Siegel-Bartel J, Rajcan-Separovic E. Submicroscopic deletions and duplications in individuals with intellectual disability detected by array-CGH. Am J Med Genet A 2005;139(3):173-85. doi: 10.1002/ajmg.a.31015 [published Online First: 2005/11/12]
22. Emsley HC, Appleton RE, Whitmore CL, Jury F, Lamb JA, Martin JE, Ollier WE, de la Morandiere KP, Southern KW, Allan SM. Variations in inflammation-related genes may be associated with childhood febrile seizure susceptibility. Seizure 2014;23(6):457-61. doi: S1059-1311(14)00072-7 [pii] 10.1016/j.seizure.2014.03.006 [published Online First: 2014/04/08]
23. Stessman HA, Xiong B, Coe BP, Wang T, Hoekzema K, Fenckova M, Kvarnung M, Gerdts J, Trinh S, Cosemans N, Vives L, Lin J, Turner TN, Santen G, Ruivenkamp C, Kriek M, van Haeringen A, Aten E, Friend K, Liebelt J, Barnett C, Haan E, Shaw M, Gecz J, Anderlid BM, Nordgren A, Lindstrand A, Schwartz C, Kooy RF, Vandeweyer G, Helsmoortel C, Romano C, Alberti A, Vinci M, Avola E, Giusto S, Courchesne E, Pramparo T, Pierce K, Nalabolu S, Amaral DG, Scheffer IE, Delatycki MB, Lockhart PJ, Hormozdiari F, Harich B, Castells-Nobau A, Xia K, Peeters H, Nordenskjold M, Schenck A, Bernier RA, Eichler EE. Targeted sequencing identifies 91 neurodevelopmental-disorder risk genes with autism and developmental-disability biases. Nat Genet 2017;49(4):515-26. doi: 10.1038/ng.3792 [published Online First: 2017/02/14]

24. Chu TT, Liu Y. An integrated genomic analysis of gene-function correlation on schizophrenia susceptibility genes. J Hum Genet 2010;55(5):285-92. doi: jhg201024 [pii] 10.1038/jhg.2010.24 [published Online First: 2010/03/27]
25. Backx L, Ceulemans B, Vermeesch JR, Devriendt K, Van Esch H. Early myoclonic encephalopathy caused by a disruption of the neuregulin-1 receptor ErbB4. Eur J Hum Genet 2009;17(3):378-82. doi: ejhg2008180 [pii] 10.1038/ejhg.2008.180 [published Online First: 2008/10/16]
26. Li KX, Lu YM, Xu ZH, Zhang J, Zhu JM, Zhang JM, Cao SX, Chen XJ, Chen Z, Luo JH, Duan S, Li XM. Neuregulin 1 regulates excitability of fast-spiking neurons through Kv1.1 and acts in epilepsy. Nat Neurosci 2011;15(2):267-73. doi: 10.1038/nn.3006 [published Online First: 2011/12/14]
27. Weissflog L, Scholz CJ, Jacob CP, Nguyen TT, Zamzow K, Gross-Lesch S, Renner TJ, Romanos M, Rujescu D, Walitza S, Kneitz S, Lesch KP, Reif A. KCNIP4 as a candidate gene for personality disorders and adult ADHD. Eur Neuropsychopharmacol 2013;23(6):436-47. doi: S0924-977X(12)00214-3 [pii] 10.1016/j.euroneuro.2012.07.017 [published Online First: 2012/09/18]
28. Hills LB, Masri A, Konno K, Kakegawa W, Lam AT, Lim-Melia E, Chandy N, Hill RS, Partlow JN, Al-Saffar M, Nasir R, Stoler JM, Barkovich AJ, Watanabe M, Yuzaki M, Mochida GH. Deletions in GRID2 lead to a recessive syndrome of cerebellar ataxia and tonic upgaze in humans. Neurology 2013;81(16):1378-86. doi: WNL.0b013e3182a841a3 [pii] 10.1212/WNL.0b013e3182a841a3 [published Online First: 2013/10/01]
29. Seeburg DP, Pak D, Sheng M. Polo-like kinases in the nervous system. Oncogene 2005;24(2):292-8. doi: 1208277 [pii] 10.1038/sj.onc.1208277 [published Online First: 2005/01/11]
30. Sun H, Kosaras B, Klein PM, Jensen FE. Mammalian target of rapamycin complex 1 activation negatively regulates Polo-like kinase 2-mediated homeostatic compensation following neonatal seizures. Proc Natl Acad Sci U S A 2013;110(13):5199-204. doi: 10.1073/pnas.1208010110 [published Online First: 2013/03/13]
31. Teoh JJ, Iwano T, Kunii M, Atik N, Avriyanti E, Yoshimura SI, Moriwaki K, Harada A. BIG1 is required for the survival of deep layer neurons, neuronal polarity, and the formation of axonal tracts between the thalamus and neocortex in developing brain. PLoS One 2017;12(4):e0175888. doi: 10.1371/journal.pone.0175888 [published Online First: 2017/04/18]
32. Euro E-RESC, Epilepsy Phenome/Genome P, Epi KC. De novo mutations in synaptic transmission genes including DNM1 cause epileptic encephalopathies. Am J Hum Genet 2014;95(4):360-70. doi: 10.1016/j.ajhg.2014.08.013 [published Online First: 2014/09/30]
33. Lesca G, Rudolf G, Labalme A, Hirsch E, Arzimanoglou A, Genton P, Motte J, de Saint Martin A, Valenti MP, Boulay C, De Bellescize J, Keo-Kosal P, Boutry-Kryza N, Edery P, Sanlaville D, Szepetowski P. Epileptic encephalopathies of the Landau-Kleffner and continuous spike and waves during slow-wave sleep types: Genomic dissection makes the link with autism. Epilepsia 2012;53(9):1526-38. doi: 10.1111/j.1528-1167.2012.03559.x [published Online First: 2012/06/29]
34. Bacchelli E, Ceroni F, Pinto D, Lomartire S, Giannandrea M, D'Adamo P, Bonora E, Parchi P, Tancredi R, Battaglia A, Maestrini E. A CTNNA3 compound heterozygous deletion implicates a role for alphaT-catenin in susceptibility to autism spectrum disorder. J Neurodev Disord 2014;6(1):17. doi: 10.1186/1866-1955-6-17 1866-1955-6-17 [pii] [published Online First: 2014/07/23]
35. Meier S, Strohmaier J, Breuer R, Mattheisen M, Degenhardt F, Muhleisen TW, Schulze TG, Nothen MM, Cichon S, Rietschel M, Wust S. Neuregulin 3 is associated with attention deficits in schizophrenia and bipolar disorder. Int J Neuropsychopharmacol 2013;16(3):549-56. doi: S1461145712000697 [pii] 10.1017/S1461145712000697 [published Online First: 2012/07/27]
36. Reggiani C, Coppens S, Sekhara T, Dimov I, Pichon B, Lufin N, Addor MC, Belligni EF, Digilio MC, Faletra F, Ferrero GB, Gerard M, Isidor B, Joss S, Niel-Butschi F, Perrone MD, Petit F, Renieri A, Romana S, Topa A, Vermeesch JR, Lenaerts T, Casimir G, Abramowicz M, Bontempi G, Vilain C, Deconinck N, Smits G. Novel promoters and coding first exons in DLG2 linked to developmental disorders and intellectual disability. Genome Med 2017;9(1):67. doi: 10.1186/s13073-017-0452-y [published Online First: 2017/07/21]
37. Mejias R, Adamczyk A, Anggono V, Niranjan T, Thomas GM, Sharma K, Skinner C, Schwartz CE, Stevenson RE, Fallin MD, Kaufmann W, Pletnikov M, Valle D, Huganir RL, Wang T. Gain-of-function glutamate receptor interacting protein 1 variants alter GluA2 recycling and surface distribution in patients

with autism. Proc Natl Acad Sci U S A 2011;108(12):4920-5. doi: 1102233108 [pii] 10.1073/pnas.1102233108 [published Online First: 2011/03/09]
38. Trotman M, Barad Z, Guevremont D, Williams J, Leitch B. Changes in the GRIP 1&2 scaffolding proteins in the cerebellum of the ataxic stargazer mouse. Brain Res 2014;1546:53-62. doi: 10.1016/j.brainres.2013.12.027 [published Online First: 2014/01/02]
39. Ishiyama S, Schmidt H, Cooper BH, Brose N, Eilers J. Munc13-3 superprimes synaptic vesicles at granule cell-to-basket cell synapses in the mouse cerebellum. J Neurosci 2014;34(44):14687-96. doi: 10.1523/JNEUROSCI.2060-14.2014 [published Online First: 2014/10/31]
40. Yu W, Du Y, Zou Y, Wang X, Stephani U, Lu Y. Smad anchor for receptor activation contributes to seizures in temporal lobe epilepsy. Synapse 2017;71(3) doi: 10.1002/syn.21957 [published Online First: 2016/12/31]
41. Weng JY, Lin YC, Lien CC. Cell type-specific expression of acid-sensing ion channels in hippocampal interneurons. J Neurosci 2010;30(19):6548-58. doi: 30/19/6548 [pii] 10.1523/JNEUROSCI.0582-10.2010 [published Online First: 2010/05/14]
42. Lee JR. Protein tyrosine phosphatase PTPRT as a regulator of synaptic formation and neuronal development. BMB Rep 2015 doi: 3148 [pii] [published Online First: 2015/03/10]
43. Kang H, Han KA, Won SY, Kim HM, Lee YH, Ko J, Um JW. Slitrk Missense Mutations Associated with Neuropsychiatric Disorders Distinctively Impair Slitrk Trafficking and Synapse Formation. Front Mol Neurosci 2016;9:104. doi: 10.3389/fnmol.2016.00104 [published Online First: 2016/11/05]
